# Supplementary material for: Transmembrane signaling by a synthetic receptor in artificial cells
Source: Nat Commun. 2023 Mar 24;14:1646. doi: 10.1038/s41467-023-37393-0 (PMC10039019; doi:10.1038/s41467-023-37393-0)
Supplement: Supplementary file 1 — Supplementary Information [file 41467_2023_37393_MOESM1_ESM.pdf]

# Transmembrane signaling by a synthetic receptor in artificial cells

Ane Bretschneider Søgaard[1, 2], Andreas Bøtker Pedersen[1], Kaja B. Løvschall[1], Pere M. Marcet [1], Josefine H. Jakobsen[1], Leila Džabbarova[1], Line F. Nielsen[1], Sandra Stevanovic[1], Raoul Walther[1] and Alexander N. Zelikin[1, 2 *]

[1]*Department of Chemistry, Aarhus University, Aarhus C, Denmark*

[2]*iNano Interdisciplinary Nanoscience Center, Aarhus University, Aarhus C, Denmark*

*Correspondence and requests for materials should be addressed to A.N.Z*
*(email: zelikin@chem.au.dk)*

# Supporting information

# Contents

1 Supplemental Figures ........ 5
1.1 Supplemental Figure 1 ........ 5
1.2 Supplemental Figure 2 ........ 5
1.3 Supplemental Figure 3 ........ 6
1.4 Supplemental Figure 4 ........ 6
2 Synthesis overview ........ 7
2.1 Synthesis of receptor scaffold ........ 7
2.2 Synthesis of Phos-EAR ........ 7
2.3 Synthesis of Glu-EAR ........ 7
2.4 Synthesis of Gus-EAR ........ 8
3 Supplemental methods ........ 9
3.1 Synthesis protocols ........ 9
3.1.1 General synthesis method A – TBS group removal ........ 9
3.1.2 General synthesis method B – carbamate formation ........ 10
3.1.3 General synthesis method C – trityl group removal ........ 10
3.1.4 Synthesis of 1 ........ 11
3.1.5 Synthesis of 2 ........ 12
3.1.6 Synthesis of 3 ........ 13
3.1.7 Synthesis of 4 ........ 14
3.1.8 Synthesis of 5 ........ 15
3.1.9 Synthesis of 6 ........ 16
3.1.10 Synthesis of 7 ........ 17
3.1.11 Synthesis of 8 ........ 18
3.1.12 Synthesis of 9 ........ 19
3.1.13 Benzyl deprotection screening data ........ 19
3.1.14 Synthesis of S1 ........ 23
3.1.15 Synthesis of 10 ........ 24
3.1.16 Synthesis of S2 ........ 25
3.1.17 Synthesis of S3 ........ 26
3.1.18 Synthesis of S4 ........ 27
3.1.19 Synthesis of 11 ........ 28
3.1.20 Synthesis of 12 ........ 29
3.1.21 Synthesis of S5 ........ 30

3.1.22 Synthesis of 13 ........ 31
3.1.23 Synthesis of S6 ........ 32
3.1.24 Synthesis of S7 ........ 33
3.1.25 Synthesis of S8 ........ 33
3.1.26 Synthesis of 14 ........ 34
3.1.27 Synthesis of 15 ........ 35
3.1.28 Synthesis of S9 ........ 36
3.1.29 Synthesis of S10 ........ 37
3.1.30 Synthesis of S11 ........ 38
3.1.31 Synthesis of S12 ........ 39
3.2 Protocols for secondary messenger release studies (HPLC) ........ 40
3.2.1 Phos-EAR ........ 40
3.2.2 Glu-EAR ........ 40
3.2.3 Gus-EAR ........ 40
3.2.4 Effect of $C_{18}$ anchor on GLU mediated cleavage (supplemental figure 1) ........ 40
3.3 NMR spectra ........ 41
3.3.1 NMR compound 1 ........ 41
3.3.2 NMR compound 2 ........ 42
3.3.3 NMR compound 3 ........ 43
3.3.4 NMR compound All-4 ........ 44
3.3.5 NMR compound All-5 ........ 45
3.3.6 NMR compound All-6 ........ 46
3.3.7 NMR compound All-7 ........ 47
3.3.8 NMR compound 8 ........ 47
3.3.9 NMR compound S1 ........ 48
3.3.10 NMR compound 10 ........ 49
3.3.11 NMR compound S2 ........ 50
3.3.12 NMR compound S4 ........ 51
3.3.13 NMR compound 11 ........ 51
3.3.14 NMR compound S5 ........ 52
3.3.15 NMR compound 13 ........ 53
3.3.16 NMR compound S6 ........ 54
3.3.17 NMR compound S8 ........ 55
3.3.18 NMR compound 14 ........ 55

3.3.19 NMR compound S9 ..... 56
3.3.20 NMR compound S10 ..... 57
3.3.21 NMR compound S11 ..... 58
3.4 HPLC chromatograms ..... 59
3.4.1 Phos-EAR (9) ..... 59
3.4.2 Glu-EAR (12) ..... 59
3.4.3 Gus-EAR (15) ..... 60

# 1 Supplemental Figures

## 1.1 Supplemental Figure 1

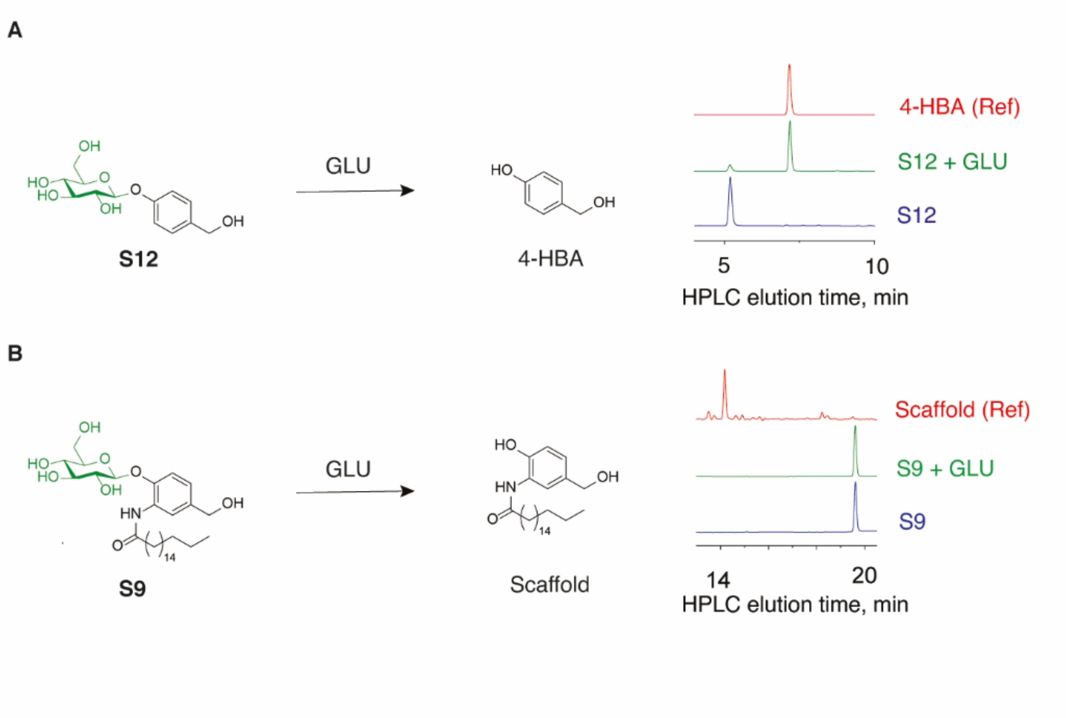


**Supplemental Figure 1. Effect of the $C_{18}$ anchor on GLU mediated cleavage.** HPLC data illustrating the cleavage of the glucosidase (GLU) specific trigger moiety without **(A)** and with **(B)** the $C_{18}$ anchor present in the molecule. 4-HBA = 4-hydroxybenzyl alcohol, Scaffold = 4-hydroxybenzyl alcohol with $C_{18}$ anchor. For more detailed protocol see section 3.2.4.

## 1.2 Supplemental Figure 2

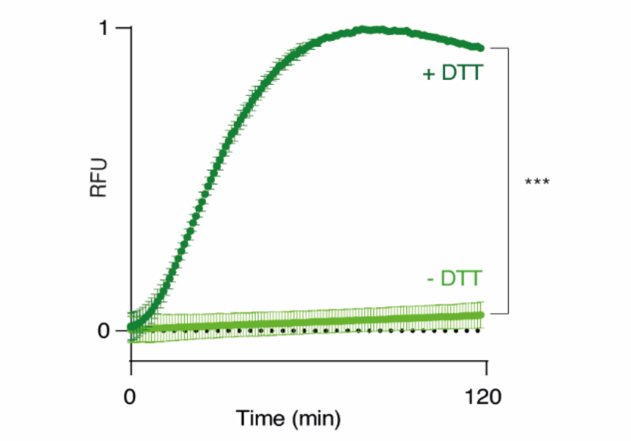


**Supplemental Figure 2. Investigating activity state of the papain stock.** Kinetic data illustrating evolution of fluorescence in suspensions that contain a disulfide-based zymogen of papain and its specific fluorogenic substrate (Arg-AMC); with (dark green) and without (light green) addition of DTT. The data is expressed relative to the max value +DTT sample of each independent replicate. the data are based on three independent experiments with triplicates (N=3, n=3) and presented as mean ± SD. RFU = relative fluorescence expressed in arbitrary units; statistical significance at endpoint was calculated via statistical significance at endpoint was calculated via an unpaired two-tailed t-test, *** $p<0.001$ (95% confidence interval).

## 1.3 Supplemental Figure 3

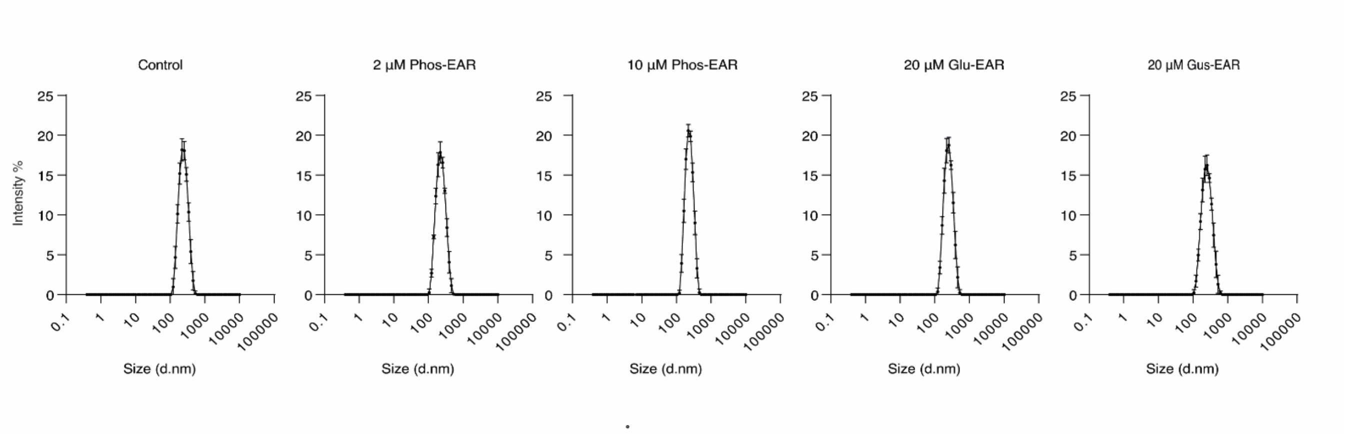


**Supplemental Figure 3. DLS measurements of liposome samples (diluted 100 times) with and without EAR.** Control; Z-average = 230.5 nm and PDI = 0.069. 2µM Phos-EAR; Z-average = 215.2 nm and PDI = 0.084. 10 µM Phos-EAR; Z-average = 227.3 nm and PDI = 0.049. 20 µM Glu-EAR; Z-average = 238.3 nm and PDI = 0.069. 10 µM Phos-EAR; Z-average = 236.5 nm and PDI = 0.105. Z-average = cumulative average size, PDI = polydispersity index, d.nm = nanometer size in diameter.

## 1.4 Supplemental Figure 4

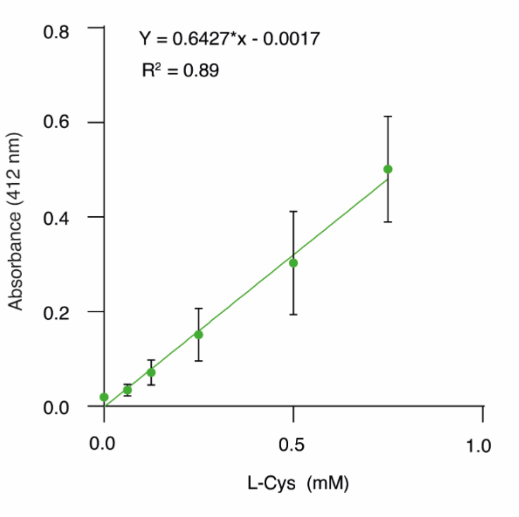


**Supplemental Figure 4. DTNB standard curve.** The standard curve is prepared using different concentrations of L-Cys. The results represent three independent replicates with triplicates (N=3, n=3). and presented as mean ± SD

# 2 Synthesis overview

## 2.1 Synthesis of receptor scaffold

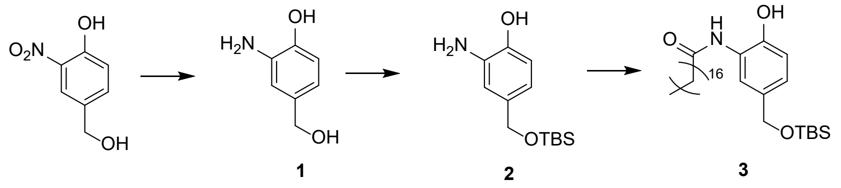


## 2.2 Synthesis of Phos-EAR

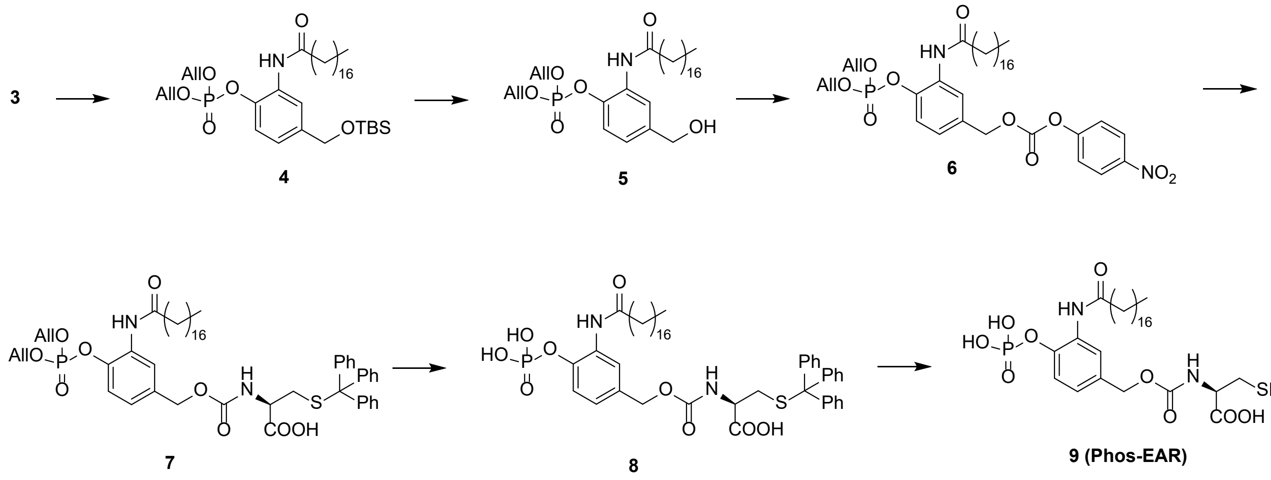


## 2.3 Synthesis of Glu-EAR

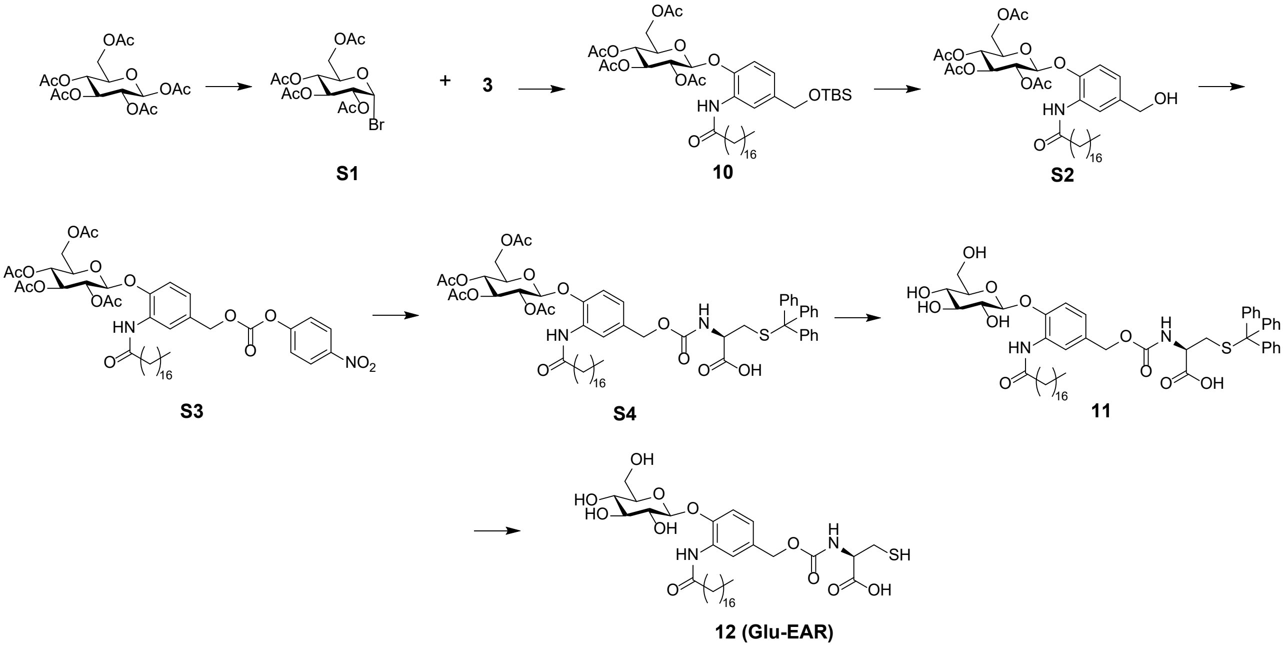

## 2.4 Synthesis of Gus-EAR

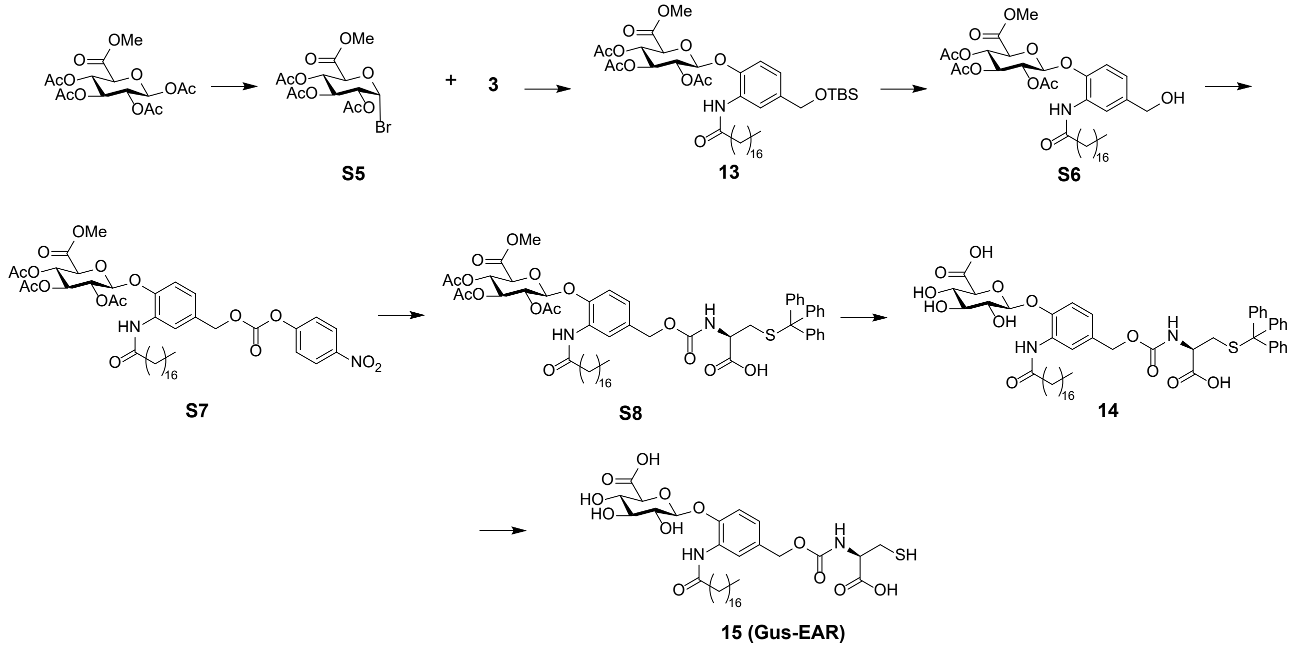

# 3 Supplemental methods

**General information on chemical experiments.** All chemicals and solvents were acquired from Sigma Aldrich and used without further purification unless otherwise stated. *N,N*-Dimethylformamide (DMF), trimethylamine (TEA), and methanol were obtained in anhydrous state. The solvents acetonitrile, dichloromethane, and tetrahydrofurane (THF) were dried over aluminum oxide using a MBraun SP800 purification system. The deuterated solvents used for $^{1}$H-NMR and $^{13}$C-NMR analysis were purchased from EurisoTop. **Thin layer chromatography** (TLC) analyses were performed using silica-coated aluminum foil plates (Merck Kieselgel 60 F254) and analyzed by visualizing either by UV irradiation and/or staining with $KMnO_4$. High purity grade silica gel (w/Ca, ~0.1%, 230-400 mesh particle size, 60Å pore size) was the stationary phase, which was also used for silica column chromatography. **NMR** spectra were recorded on a Bruker AVANCE III HD spectrometer operating at $^{1}$H-NMR 400 MHz or $^{13}$C-NMR 101 MHz. The spectra were referenced to the solvent peak. $CDCl_3$ ($\delta_H$ 7.26 ppm, $\delta_C$ 77.16 ppm), $CD_3OD$ ($\delta_H$ 3.31 ppm, $\delta_C$ 49.00 ppm), DMSO-$d_6$ ($\delta_H$ 2.50 ppm, $\delta_C$ 39.52 ppm). **High-resolution mass spectrometry (HR-MS)** was performed using a Bruker Micromass LC-TOF spectrometer with electrospray ionization (ESI) and analyzed with Bruker DataAnalysis.

**High-performance liquid chromatography (HPLC)** experiments were conducted with an Agilent 1260 Infinity II connected to an EC-C18 column with particle size of 2.7 µm, length of 100 mm and diameter of 4.6 mm (flow rate: 0.4 mL/min). The mobile phase was a combination of ultrapure water with trifluoroacetic acid (TFA, 0.1% v/v%, eluent A) and HPLC grade acetonitrile with TFA (0.1% v/v%, eluent B). HPLC experiments were performed with following methods; **HPLC method A:** the mobile was initially 10% B and was gradually increased to 100% B over 20 minutes. The eluent was then kept at 100% B for 8 minutes until the analysis was stopped. UV signals were detected at wavelengths $\lambda$ = 220 nm and $\lambda$ = 254 nm. **HPLC method B:** started at 5% eluent B content and the eluent B content was gradually increased to 100% within 18 minutes and kept at 100% content for additionally 13 minutes (total time of 31 minutes). UV was measured with the wavelengths $\lambda$ = 210 nm and $\lambda$ = 254 nm. Preparative HPLC purifications were carried out using either a ZORBAX C18 column with a particle size of 5 µm, a length of 250 mm and an internal diameter of 9.4 mm (flow rate: 10 mL/min), or a ZORBAX XDB-C8 column with a particle size of 5 µm, a length of 250 mm and an internal diameter of 9.4 mm (flow rate: 10 mL/min).

**General information on software and code.** The following software was used for data collection and/or analysis: Agilent Openlab CDS Acquisition 2.5 (HPLC); Astra 7.3.2 (SEC); Gen5 3.10 (plate reader); OriginPro 2018 (data processing and plotting); Graphpad Prism v. 9 (data processing, plotting, statistical analsysi); Bruker Compass DataAnalysis 4.2 (MS data); MestReNova v14.2.0 (NMR).

## 3.1 Synthesis protocols

### 3.1.1 General synthesis method A – TBS group removal

In a flame dried flask, TBS-protected benzyl alcohol (1 equiv.) was dissolved in ethanol and cooled to 0 °C. Conc. hydrochloric acid (10 equiv.) was added and the solution was stirred for an hour. Upon

completion, the solution was neutralized with sodium bicarbonate. The mixture was extracted three times with $CH_2Cl_2$ and the combined organic layers were dried over magnesium sulfate. Upon drying *in vacuo*, the benzyl alcohol compound was obtained. Without further purification, it was directly used in next step.

### 3.1.2 General synthesis method B – carbamate formation

The carbonate compound (1 equiv.) was dissolved in dry $CH_2Cl_2$ and added dropwise to a stirring suspension of *S*-trityl-L-cysteine (1.6 equiv.) and triethylamine (3 equiv.) in dry $CH_2Cl_2$ under an atmosphere of argon at 0 °C. The mixture was allowed to heat to room temperature and was stirred over night before full conversion was observed on TLC. $CH_2Cl_2$ was evaporated under reduced pressure, the resulting crude mixture was absorbed onto Celite® and purified by silica column chromatography yielding the carbamate.

### 3.1.3 General synthesis method C – trityl group removal

*S*-trityl-protected receptor (1 equiv.) was dissolved in dry $CH_2Cl_2$ and the solution was cooled to 0 °C under an atmosphere of $N_2$. To the mixture was added $(i\text{-Pr})_3SiH$ (2 equiv.) and trifluoroacetic acid (TFA) (50 equiv.). The reaction mixture was then allowed to heat to room temperature and was stirred for 2 hours until full conversion was observed. The solvent was removed under reduced pressure and the resulting crude mixture was purified by silica column chromatography or preparative HPLC yielding the desired receptor compound.

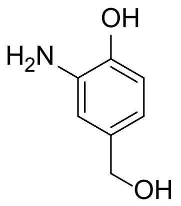


**1**

### 3.1.4 Synthesis of 1

To a dry round-bottom flask with a stir bar, 4-hydroxy-3-nitrobenzyl alcohol (2 g, 11.8 mmol) was added and dissolved in EtOAc/MeOH (150:15 mL). To this, Pd/C (200 mg) was added and the reaction mixture was purged by bubbling $H_2$ gas (via a balloon) through the reaction mixture for 5 min. The reaction was then maintained under a balloon of $H_2$ for 15-20 hours. After confirming the completion of reaction by TLC, the $H_2$ balloon was carefully detached and reacting mixture was purged with $N_2$. The contents were filtered over a bed of Celite. The filtrate was then concentrated by evaporating solvents using a rotatory evaporator to yield **1** (1.609 g, 11.6 mmol, 98%) as brown crystals.

**$^1$H-NMR** (400 MHz, $CD_3OD$) $\delta_H$ (ppm) 6.76 (d, $J$ = 2.1 Hz, 1H), 6.66 (d, $J$ = 8.1 Hz, 1H), 6.58 (dd, $J$ = 8.0, 2.1 Hz, 1H), 4.41 (s, 2H).

**$^{13}$C-NMR** (101 MHz, $CD_3OD$) $\delta_C$ (ppm) 146.0, 136.1, 134.1, 119.3, 116.8, 115.3, 65.4

**HR-MS (ESI)**: $[C_7H_{10}NO_2]^+$ calcd. 140.0707, found 140.0714.

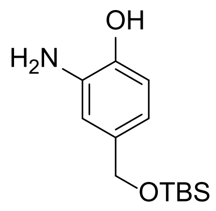

**2**

### 3.1.5 Synthesis of 2

**1** (783 mg, 5.63 mmol, 1 equiv.) was dissolved in DMF (27 mL) under a $N_2$ atmosphere. Imidazole (0.68 mL, 10.1 mmol, 1.8 equiv.) and 4-dimethylaminopyridine (DMAP) (172 mg, 1.4 mmol, 0.25 equiv.) was added to the solution. *Tert*-butyldimethylsilyl chloride (TBDMSCl) (1.02 g, 6.8 mmol, 1.2 equiv.) was dissolved in DMF (19 mL) and added dropwise to the reaction mixture. The reaction stood for 2 hours before it was completed as shown by TLC. Following completion the reaction was diluted with $CH_2Cl_2$, washed with sat. ammonium chloride (3 x 50 mL) and brine (2 x 50 mL). The organic layers were dried with sodium sulfate filtered and concentrated *in vacuo*. The product was isolated through flash column chromatography (EtOAc/Pentane 1:3) as a red solid (980 mg, 3.9 mmol, 69%).

**$^{1}$H-NMR** (400 MHz, $CDCl_3$) $\delta_H$ (ppm) 6.74 (d, $J$ = 2.1 Hz, 1H), 6.70-6.58 (m, 2H), 4.59 (s, 2H), 0.93 (s, 9H), 0.09 (s, 6H).

**$^{13}$C-NMR** (101 MHz, $CDCl_3$) $\delta_C$ (ppm) 143.3, 134.6, 134.4, 117.7, 115.5, 115.2, 65.1, 26.2, 18.6, (-5.0).

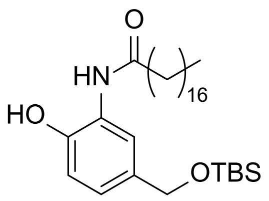

**3**

### 3.1.6 Synthesis of 3

In a round bottom flask, **2** (46 mg, 0.18 mmol, 1 equiv.) and stearic acid (52 mg, 0.18 mmol, 1 equiv.) were placed in anhydrous $CH_2Cl_2$ (1.5 mL) at room temperature. The mixture was placed under stirring in a $N_2$ atmosphere. Diisopropylethylamine (DIPEA) (0.035 mL, 0.2 mmol, 1.1 equiv.) was added, followed by the addition of benzotriazol-1-yloxytris(dimethylamino)phosphonium hexafluorophosphate (BOP) (89 mg, 0.2 mmol, 1.1 equiv.) dissolved in 0.5 mL of $CH_2Cl_2$. The mixture was stirred for 12 hours at 20 °C. The solvent was removed under vacuum and the crude residue purified by chromatography on a silica gel column using EtOAc/Pentane 1:9 to 1:7 as eluent (77 mg, 0.5 mmmol, 81%).

**$^1$H-NMR** (400 MHz, $CDCl_3$) $\delta_H$ (ppm) 8.80 (s, 1H), 7.38 (b.s., 1H), 7.05 (dd, $J$ = 8.3, 2.2 Hz, 1H), 6.97 (d, $J$ = Hz, 1H), 6.93 (d, $J$ = 2.2 Hz), 4.63 (s, 2H), 2.45 (t, $J$ = 7.6 Hz, 2H), 1.75 (t, $J$ = 7.3 Hz, 2H), 1.25 (s, 28H), 0.93 (s, 9H), 0.88 (t, $J$ = 7.3 Hz, 3H), 0.09 (s, 6H).

**$^{13}$C-NMR** (101 MHz, $CDCl_3$) $\delta_C$ (ppm) 173.8, 148.0, 133.7, 125.4, 125.3, 120.1, 120.0, 64.5, 37.2, 32.1, 29.9, 29.8, 29.8, 29.8, 29.6, 29.5, 29.3, 26.1, 25.9, 22.8, 18.6, 14.3, (-5.1).

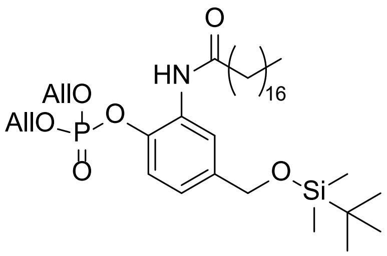

**4**

### 3.1.7 Synthesis of 4

In a flame dried flask, Phosphorus(V) oxychloride (0.045 mL, 0.50 mmol, 1.25 equiv.) was dissolved in dry $CH_2Cl_2$ (5.0 mL) and stirred at 0 °C. **3** (0.206 g, 0.40 mmol, 1 equiv.) and TEA (0.11 mL, 0.79 mmol, 2 equiv.) were added slowly through syringe. The reaction solution was stirred at room temperature for 1 hour until full conversion of the phenol was observed by TLC. The reaction was again cooled to 0 °C and allyl alcohol (0.16 mL, 2.4 mmol, 6 equiv.) and TEA (0.22 mL, 1.59 mmol, 4 equiv.) were slowly added. The reaction was left to stir for 3 hours until full conversion of the chlorophosphate was observed by TLC. The mixture was diluted with $CH_2Cl_2$, washed with sat. $NaHCO_3$, 0.5 M $KHSO_4$, water, and sat. $NaHCO_3$. The combined organic phase was dried with $Na_2SO_4$, filtered, and concentrated under reduced pressure. After purification by flash column chromatography (pentate:EtOAc 9:1 to 7:1) the product was isolated as a white solid (176 mg, 0.26 mmol, 65%).

**$^1$H-NMR** (400 MHz, $CDCl_3$) $\delta_H$ (ppm) 8.25 (s, 1H), 8.15 (s, 1H), 7.17 (d, *J* = 8.4 Hz, 1H), 7.09 (d, *J* = 8.4 Hz, 1h), 5.91 (ddt, *J* = 16.3, 10.7, 5.8 Hz, 2H), 5.37 (d, *J* = 17.1 Hz, 2H), 5.28 (d, *J* = 9.2 Hz, 2H), 4.70 (s, 2H), 4.67-4.57 (m, 4H), 2.37 (t, *J* = 7.6 Hz, 2H), 1.72 (t, *J* = 7.2 Hz, 2H), 1.25 (s, 28H), 0.93 (s, 9H), 0.88 (t, *J* = 6.9 Hz, 3H), 0.09 (s, 6H).

**$^{13}$C-NMR** (101 MHz, $CDCl_3$) $\delta_C$ (ppm) 171.7, 139.6, 138.7, 131.9, 131.8, 129.6, 122.0, 120.5, 119.3, 69.5, 69.5, 64.6, 38.0, 32.0, 29.8, 29.8, 29.6, 29.5, 29.5, 29.4, 26.0, 25.7, 22.8, 18.5, 14.2, -5.2.

**HR-MS (ESI)**: $[C_{37}H_{66}NO_6PSi+H]^+$ calcd.: 680.4470, found: 680.4490. $[C_{37}H_{66}NO_6PSi+Na]^+$ calcd.: 702.4290, found: 702.4308.

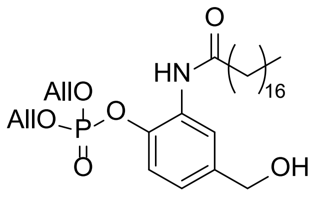


**5**

### 3.1.8 Synthesis of 5

According to **General synthesis method A**, **4** (340 mg, 0.50 mmol, 1 equiv.) and conc. hydrochloric acid (0.43 mL, 5.0 mmol, 10 equiv.) in ethanol (5.3 mL) was reacted to produce **5**, which was obtained as a white solid (280 mg, 0.49 mmol, 99%) by removing solvent *in vacuo*. Without further purification, it was directly used in next step.

**$^{1}$H-NMR** (400 MHz, $CDCl_3$) $\delta_H$ (ppm) 8.30 (s, 1H), 8.21 (s, 1H), 7.18 (d, *J* = 8.3 Hz, 1H), 7.09 (d, *J* = 8.4 Hz, 1H), 5.90 (ddt, *J* = 16.3, 10.7, 5.8 Hz, 2H), 5.37 (d, *J* = 17.1 Hz, 2H), 5.28 (d, *J* = 10.4 Hz, 2H), 4.66-4.59 (m, 6H), 2.37 (t, *J* = 7.6 Hz, 2H), 1.71 (t, *J* = 7.4 Hz, 2H), 1.25 (s, 28H), 0.87 (t, *J* = 6.9 Hz, 3H).

**$^{13}$C-NMR** (101 MHz, $CDCl_3$) $\delta_C$ (ppm) 171.9, 139.2, 131.8, 131.8, 129.8, 123.0, 121.6, 120.8, 119.4, 69.5, 69.5, 64.7, 37.9, 32.0, 29.8, 29.6, 29.5, 29.5, 29.4, 25.8, 25.6, 22.8, 14.2.

**HR-MS (ESI)**: $[C_{31}H_{57}NO_6P+H]^+$ calcd.: 566.3605, found: 566.3647. $[C_{31}H_{57}NO_6P+Na]^+$ calcd.: 588.3425, found: 588.3460.

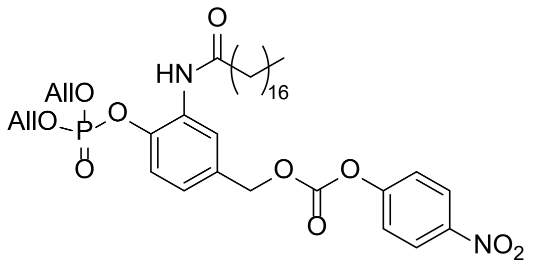


**6**

### 3.1.9 Synthesis of 6

The benzyl alcohol **5** (50 mg, 0.088 mmol 1 equiv.) was added to a flame dried flask and dissolved in dry $CH_2Cl_2$ (0.4 mL) under an atmosphere of argon and cooled to 0 °C. Triethylamine (0.037 mL, 0.27 mmol, 3 equiv.) was added to the solution. After 5 minutes, a solution of 4-nitrophenyl chloroformate (0.025 g, 0.12 mmol, 1.4 equiv.) in dry $CH_2Cl_2$ (0.4 mL) was added dropwise to the mixture and the resulting solution was allowed to warm to room temperature. The reaction mixture was stirred overnight and full conversion was observed by TLC. The crude product was evaporated onto Celite®, and purified by silica column chromatography (Pentane:EtOAc 4:1 to 2:1) and the carbonate **6** was isolated as a white solid (46 mg, 0.063 mmol, 71%).

**$^1$H-NMR** (400 MHz, $CDCl_3$) $\delta_H$ (ppm) 8.41 (s, 1H), 8.30-8.25 (m, 3H), 7.39 (d, $J$ = 9.2 Hz, 2H), 7.24 (s, 1H), 7.13 (d, $J$ = 8.3 Hz, 1H), 5.93 (ddt, $J$ = 16.3, 10.7, 5.8 Hz, 2H), 5.39 (dd, $J$ = 17.1, 1.3 Hz, 2H), 5.29 (dd, $J$ = 10.5, 1.0 Hz, 2H), 5.25 (s, 2H), 4.66 (ddd, $J$ = 9.1, 4.5, 1.3 Hz, 4H), 2.40 (t, $J$ =7.6 Hz, 2H), 1.73 (t, $J$ = 7.4 Hz, 2H), 1.25 (s, 28H), 0.88 (t, $J$ = 7.2 Hz, 3H).

**$^{13}$C-NMR** (101 MHz, $CDCl_3$) $\delta_C$ (ppm) 171.9, 171.3, 155.7, 152.5, 145.4 140.1, 132.2, 131.7, 130.4, 125.4, 124.4, 123.0, 122.0, 121.0, 119.6, 70.4, 69.7, 60.5, 53.6, 38.0, 34.3, 32.1, 29.8, 29.7, 29.5, 29.5, 29.4, 25.6, 22.8, 22.5, 21.2, 14.2.

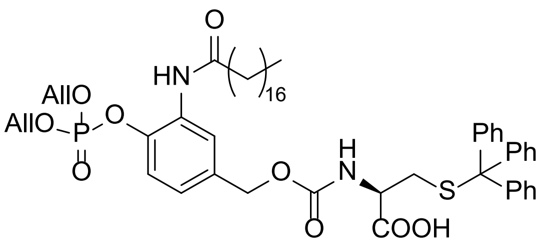


**7**

**3.1.10 Synthesis of 7**

According to **General synthesis method B**, **6** (0.020 g, 0.027 mmol, 1 equiv.) in dry $CH_2Cl_2$ (0.5 mL) was added to a stirring suspension of *S*-trityl-L-cysteine (0.016 g, 0.044 mmol, 1.6 equiv.) and triethylamine (TEA) (11 µL, 0.08 mmol, 3 equiv.) in dry $CH_2Cl_2$ (0.5 mL) and reacted overnight. Purification of **7** was performed by silica column chromatography (3-8% MeOH in $CH_2Cl_2$) yielding the desired product as a white solid (13 mg, 0.014 mmol, 50%).

**$^1$H-NMR** (400 MHz, $CD_3OD$) $\delta_H$ (ppm) 7.82 (s, 1H), 7.39-7.33 (m, 6H), 7.31-7.14 (m, 12H), 5.95 (ddt, *J* = 16.3, 11.0, 5.7 Hz, 2H), 5.37 (dd, *J* = 17.1, 1.6 Hz, 2H), 5.25 (dd, *J* = 10.5, 1.3 Hz, 2H), 5.10-4.96 (m, 2H), 4.69-4.59 (m, 4H), 4.13-4.06 (m, 1H), 2.60 (d, *J* = 6.5 Hz, 2H), 2.40 (t, *J* = 7.5 Hz, 2H), 1.69 (t, *J* = 7.4 Hz, 2H), 1.28 (s, 28H), 0.89 (t, *J* = 7.1 Hz, 3H).

**HR-MS (ESI)**: $[C_{59}H_{71}N_2O_9PS+H]^+$ calcd.: 955.4691, found: 955.4695

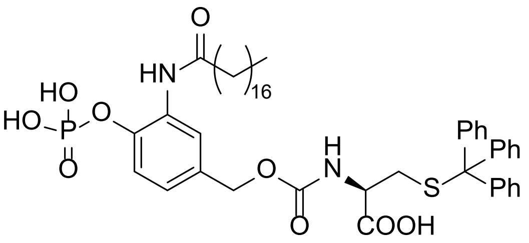


**8**

### 3.1.11 Synthesis of 8

To a flame dried flask containing **7** (4.0 mg, 4 µmol, 1 equiv.) in $CH_2Cl_2$ (0.50 mL), tetrakis(triphenylphosphine) palladium (0.5 mg, 0.4 µmol, 0.1 equiv.), and triphenylphosphine (0.2 mg, 0.8 µmol, 0.2 equiv.) under a $N_2$ atmosphere at room temperature, pyrrolidine (7 µL, 0.08 mmol, 20 equiv.) was added. The reaction stood for 1 hour until consumption of **7** was observed by TLC (EtOAC:MeOH 9:1) after which, the reaction mixture was concentrated under reduced pressure. The crude mixture was purified by flash column chromatography (EtOAc:MeOH 9:1 to EtOAc:MeOH:MeCN:$H_2O$ 7:1:1:1) and the isolated fractions were lyophilized and the desired product was isolated as the pyrrolidinium salt of **8** as a white solid (1.1 mg, 1.3 µmol, 33%).

**$^1$H-NMR** (400 MHz, DMSO-d6) $\delta_H$ (ppm) 11.27 (s, 1H), 7.97 (s, 1H), 7.37-7.18 (m, 15H), 6.96-6.87 (m, 2H), 6.53 (s, 1H), 4.95-4.82 (m, 2H), 3.75-3.67 (m, 1H), 3.02-2.90 (m, 8H), 2.42-2.37 (m, 2H), 2.22 (t, $J$ = 7.5 Hz, 2H), 1.79-1.69 (m, 8H), 1.62-1.52 (m, 2H), 1.23 (s, 28H), 0.85 (t, $J$ = 7.1 Hz, 3H).

**HR-MS (ESI)**: $[C_{48}H_{63}N_2O_9PS\text{-}H]^-$ calcd.: 873.3919, found: 873.3923.

**HPLC**: $t_r$ (**8**): 14.78 min (**HPLC method A**)

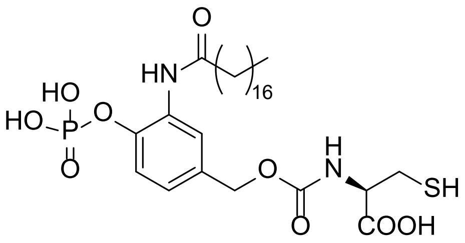

**9 (Phos-EAR)**

### 3.1.12 Synthesis of 9

According to **General synthesis method C**, **8** (1.1 mg, 1.3 µmol, 1 equiv.) was reacted with (*i*-Pr)$_3$SiH (0.5 µL, 2.5 µmol, 2 equiv.) and trifluoroacetic acid (TFA) (5 µL, 63 µmol, 50 equiv.) in $CH_2Cl_2$ (0.5 mL). After reacting for 2 hours, the crude mixture was purified by silica column chromatography (10% MeOH in EtOAc followed by EtOAc:MeOH:EtOH:$H_2O$ 6:2:1:1) yielding the desired compound **9 (EAR)** as a white solid (0.63 mg, 1.0 µmol, 79%) which was shown to be pure by HPLC.

**HPLC**: $t_r$ (**9**): 21.89 min (**HPLC method A**)

**HR-MS (ESI)**: $[C_{29}H_{49}N_2O_9PS\text{-}H]^-$ calcd.: 631.2823, found: 631.2826.

### 3.1.13 Benzyl deprotection screening data

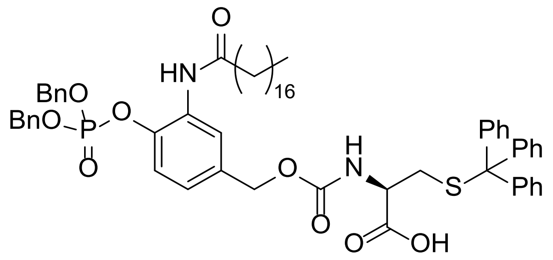


**Bn-7**

### 3.1.13.1 Deprotection conditions using TFA

**Bn-7** (8 mg, 8 µmol, 1 equiv.) was dissolved in $CH_2Cl_2$ (0.3 mL) in an inert atmosphere. The solution was cooled to 0 °C and TFA (0.3 mL) was added to the reaction and the mixture was allowed to heat to room temperature. The reaction was stopped after 2 hours and diluted with $CH_2Cl_2$. Upon termination of the reaction, solvents were evaporated under reduced pressure and a sample of the mixture of products was dissolved in water/MeCN 1:1 and filtered before MS analysis.

Mass spectrum after 2 hours:

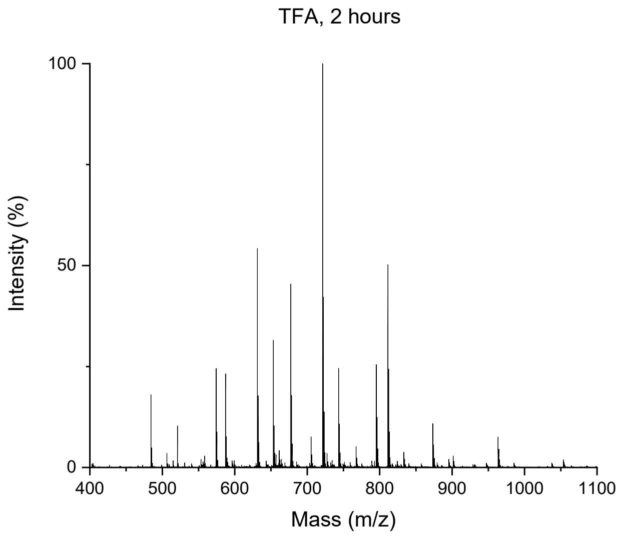


### 3.1.13.2 Deprotection conditions using TMSBr

**Bn-7** (8 mg, 8 µmol, 1 equiv.) was dissolved in MeCN (0.25 mL) under an inert atmosphere. The solution was cooled to 0 °C and trimethylsilyl bromide (2.5 µL, 0.02 mmol, 2.5 equiv.) was added to the reaction and the mixture was allowed to heat to room temperature. The reaction was stopped after 2 hours and the mixture was diluted with MeCN. Upon termination of the reaction, solvents were evaporated under reduced pressure and a sample of the mixture of products was dissolved in water/MeCN 1:1 and filtered before MS analysis.

Mass spectrum after 2 hours:

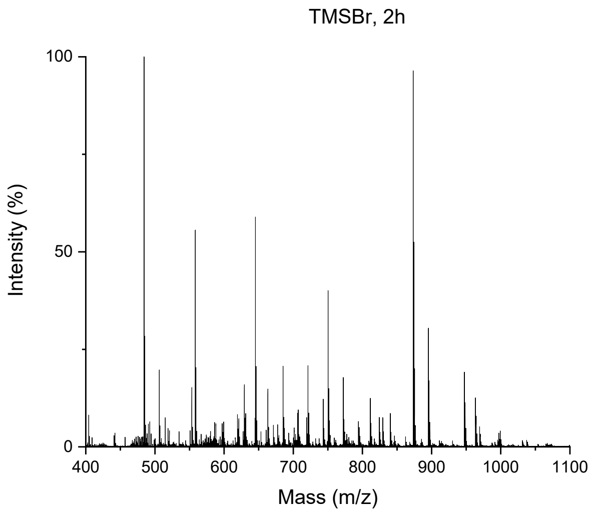


### 3.1.13.3 Allyl deprotection screening data

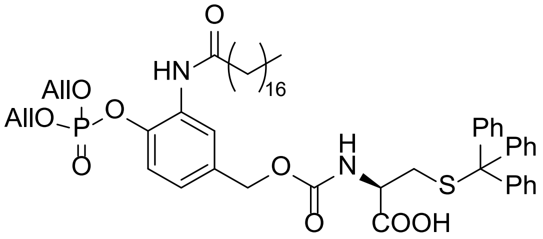


**All-7**

To **All-7** (4.0 mg, 4 µmol, 1 equiv.) in $CH_2Cl_2$ (0.20 mL), tetrakis(triphenylphosphine) palladium (0.5 mg, 0.4 µmol, 0.1 equiv.), and triphenylphosphine (0.2 mg, 0.8 µmol, 0.2 equiv.) was added under a $N_2$ atmosphere at room temperature. Pyrrolidine (7 µL, 0.08 mmol, 20 equiv.) was added to the stirring solution. The reaction was stopped after 2 hours and diluted with $CH_2Cl_2$. Upon termination of the reaction, solvents were evaporated under reduced pressure and a sample the mixture of products was dissolved in water/MeCN 1:1 and filtered before MS analysis.

Mass spectrum after 2 hours:

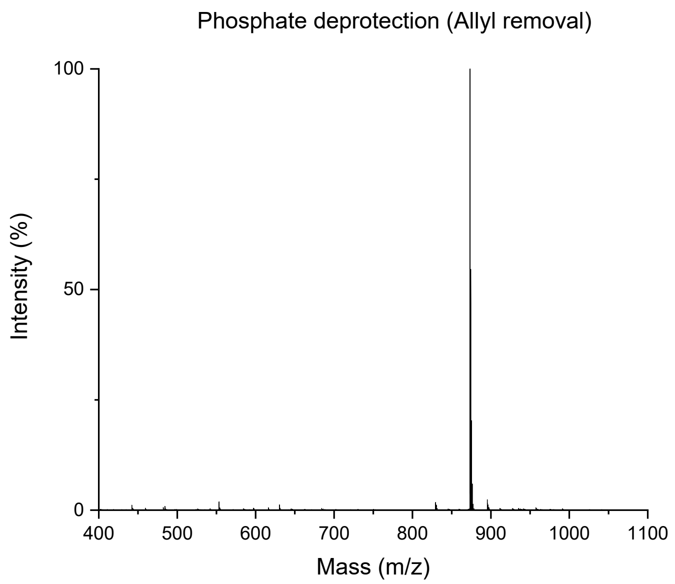
Phosphate deprotection (Allyl removal)
100
50
0
Intensity (%)
400
500
600
700
800
900
1000
1100
Mass (m/z)

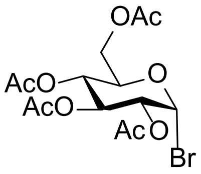


**S1**

### 3.1.14 Synthesis of S1

β-D-Glucose pentaacetate (1.0311 g, 2.6413 mmol, 1.0 equiv.) was dissolved in anhydrous $CH_2Cl_2$ (5 mL) under an atmosphere of argon and cooled to 0 °C. HBr in AcOH (33 wt. %, 10 mL, 55 mmol, 21 equiv.) was added dropwise to the solution and the reaction mixture was allowed to reach room temperature. After stirring for 3 hours the reaction mixture was poured onto ice. The organic phase was washed with ice water (x3) and saturated $NaHCO_3$ (x3). The organic layer was dried over $MgSO_4$, filtered and concentrated *in vacuo* to give the acetylated α-glucosyl bromide **S1** (1.1315 g, 2.7517 mmol, quantitative) as a sticky white solid.

**$^1$H NMR** (400 MHz, $CDCl_3$) $\delta_H$ (ppm) 6.60 (d, $J$ = 4.0 Hz, 1H), 5.55 (t, $J$ = 9.7 Hz, 1H), 5.15 (t, $J$ = 9.7 Hz, 1H), 4.83 (dd, $J$ = 10.1, 4.0 Hz, 1H), 4.34 – 4.26 (m, 2H), 4.15 – 4.09 (m, 1H), 2.10 – 2.02 (4s, 12H).

**$^{13}$C NMR** (101 MHz, $CDCl_3$) $\delta_C$ (ppm) 170.6, 170.0, 169.9, 169.6, 86.7, 72.2, 70.7, 70.3, 67.3, 61.1, 20.8, 20.8, 20.7, 20.7.

**HR-MS** (ESI): $[C_{14}H_{19}BrO_9 + Na]^+$ calcd. 433.0104, found 433.0102.

***R*f** (EtOAc/Pentane 2:3) 0.57.

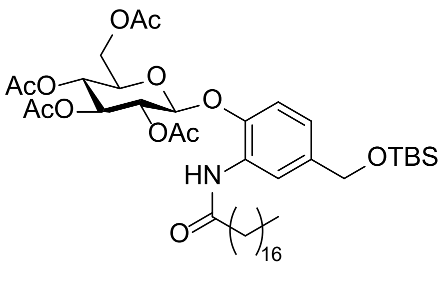


**10**

### 3.1.15 Synthesis of 10

The acetylated α-glucosyl bromide **S1** (208.6 mg, 0.5073 mmol, 1.0 equiv.), NaI (145.0 mg, 0.9674 mmol, 1.9 equiv.) and $Na_2SO_4$ (204.6 mg) was mixed in anhydrous $CH_3CN$ (2.5 mL) in a flame-dried flask, which had been evacuated and filled with argon atmosphere three times. The mixture was stirred for 30 minutes before phenol derivative **3** (345.3 mg, 0.6642 mmol, 1.3 equiv.) in anhydrous $CH_2Cl_2$ (5 mL) was added. After stirring for additional 50 minutes, $Ag_2O$ (223.8 mg, 0.9657 mmol, 1.9 equiv.) was added to the reaction mixture, which was hereafter stirred in the dark for a total of 21 hours. The crude was filtered over a plug of Celite® followed by washing with $CH_2Cl_2$. The filtrate was concentrated under reduced pressure and purified by flash column chromatography (1:4 (EtOAc/Pentane)) to yield the desired glucoside **10** (β-anomer ) (84.7 mg, 0.09962 mmol, 20 %) as a colourless oil.

**$^1$H NMR** (400 MHz, $CDCl_3$) $\delta_H$ (ppm) 8.35 (d, *J* = 2.1 Hz, 1H), 7.79 (s, 1H), 7.03 (dd, *J* = 8.3, 2.1 Hz, 1H), 6.90 (d, *J* = 8.4 Hz, 1H), 5.39-5.26 (m, 2H), 5.14 (dd, *J* = 10.0, 8.8 Hz, 1H), 5.00 (d, *J* = 7.4 Hz, 1H), 4.66 (s, 2H), 4.33 (dd, *J* = 12.4, 5.4 Hz, 1H), 4.14 (dd, *J* = 12.4, 2.3 Hz, 1H), 3.88 (ddd, *J* = 10.1, 5.4, 2.3 Hz, 1H), 2.38 (m, 2H), 2.06 (4s, 12H), 1.71 (m, 2H), 1.24 (s, 28H), 0.92 (s, 9H), 0.86 (t, *J* = 6.7 Hz, 3H), 0.08 (s, 6H).

**$^{13}$C NMR** (101 MHz, $CDCl_3$) $\delta_C$ (ppm) 172.0, 170.6, 170.4, 170.1, 169.5, 144.0, 137.3, 128.8, 121.0, 118.1, 113.8, 100.1, 72.2, 72.1, 71.5, 68.3, 64.8, 61.8, 37.9, 32.0, 29.8, 29.8, 29.7, 29.5, 29.5, 26.1, 25.8, 22.8, 21.0, 20.7, 20.7, 18.5, 14.2, (-5.1).

**HR-MS** (ESI): $[C_{45}H_{75}NO_{12}Si + H]^+$ calcd. 850.5132, found 850.5159.

***R*f** (EtOAc/Pentane 1:4) 0.48.

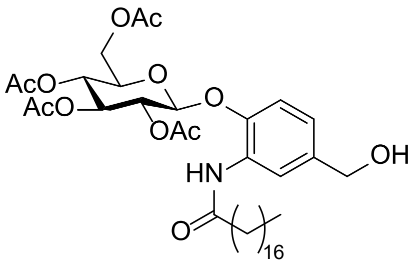

**S2**

### 3.1.16 Synthesis of S2

According to **General synthesis method A**, **10** (83 mg, 0.098 mmol, 1 equiv.) and conc. hydrochloric acid (0.10 mL, 1.2 mmol, 12 equiv.) in ethanol (3.0 mL) was reacted to produce **S2**, which was obtained by flash column chromatography (3:2 (EtOAc/Pentane)) to yield the benzyl alcohol as a sticky white solid (54.6 mg, 0.0742 mmol, 76 %).

**$^{1}$H NMR** (400 MHz, $CDCl_3$) $\delta_H$ (ppm) 8.40 (d, *J* = 2.1 Hz, 1H), 7.81 (s, 1H), 7.03 (dd, *J* = 8.4, 2.1 Hz, 1H), 6.91 (d, *J* = 8.3 Hz, 1H), 5.39 – 5.25 (m, 2H), 5.13 (t, *J* = 9.5 Hz, 1H), 5.02 (d, *J* = 7.5 Hz, 1H), 4.59 (s, 2H), 4.32 (dd, *J* = 12.4, 5.3 Hz, 1H), 4.14 (dd, *J* = 12.4, 2.3 Hz, 1H), 3.89 (ddd, *J* = 10.1, 5.4, 2.3 Hz, 1H), 2.38 (td, *J* = 7.4, 3.6 Hz, 2H), 2.26 (d, *J* = 26.2 Hz, 1H), 2.09 – 2.01 (m, 12H), 1.70 (q, *J* = 7.6 Hz, 2H), 1.24 (s, 28H), 0.86 (t, *J* = 6.7 Hz, 3H).

**$^{13}$C NMR** (101 MHz, $CDCl_3$) $\delta_C$ (ppm) 172.2, 170.6, 170.4, 170.1, 169.6, 144.4, 136.9, 128.9, 122.0, 119.2, 113.9, 99.9, 72.2, 72.0, 71.5, 68.3, 64.9, 61.8, 37.8, 32.0, 29.8, 29.7, 29.6, 29.5, 29.4, 25.8, 22.8, 21.0, 20.8, 20.7, 14.2.

**HR-MS** (ESI): $[C_{39}H_{61}NO_{12} + H]^+$ calcd. 736.4267, found 736.4294. $[C_{39}H_{61}NO_{12} + Na]^+$ calcd. 758.4086, found 758.4118. $[C_{39}H_{61}NO_{12} + K]^+$ calcd. 774.3826, found 774.3844.

***R*f** (EtOAc/Pentane 3:2) 0.32.

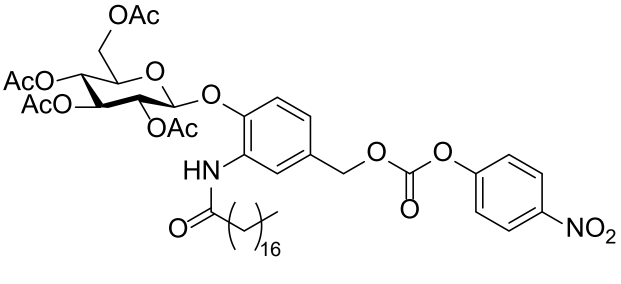


**S3**

### 3.1.17 Synthesis of S3

The benzyl alcohol **S2** (44.9 mg, 0.0610 mmol, 1 equiv.) and TEA (25 µL, 0.18 mmol, 2.9 equiv.) were dissolved in anhydrous $CH_2Cl_2$ (0.5 mL) under an atmosphere of argon and cooled to 0 °C. A solution of 4-nitrophenyl chloroformate (18.1 mg, 0.0898 mmol, 1.5 equiv.) in $CH_2Cl_2$ (0.5 mL) was added dropwise to the mixture, and the reaction mixture was allowed to reach room temperature. After stirring for 18.5 hours the reaction mixture was diluted with $CH_2Cl_2$ and the organic phase was washed with brine (x3), dried over $MgSO_4$, filtered and concentrated *in vacuo*. The carbonate derivative **S3** was used in the next step without further purification.

**HR-MS** (ESI): $[C_{46}H_{64}N_2O_{16} + H]^+$ calcd. 901.4329, found 901.4360. $[C_{46}H_{64}N_2O_{16} + Na]^+$ calcd. 923.4148, found 923.4179.

$\boldsymbol{R}_f$ (EtOAc/Pentane 1:2) 0.33.

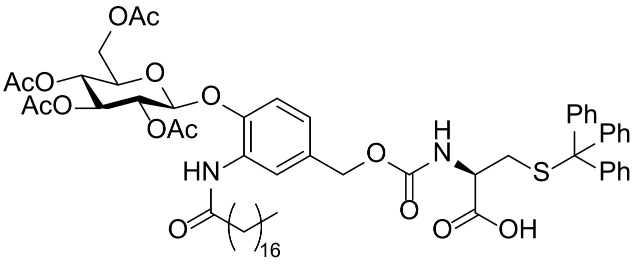


**S4**

### 3.1.18 Synthesis of S4

According to **General synthesis method B**, **S3** (0.061 mmol, 1 equiv.) in dry $CH_2Cl_2$ (0.7 mL) was added to a stirring suspension of *S*-trityl-L-cysteine (33 mg, 0.090 mmol, 1.5 equiv.) and triethylamine (TEA) (0.04 mL, 0.29 mmol, 4.7 equiv.) in dry $CH_2Cl_2$ (0.7 mL) and reacted overnight. Purification of **S4** was performed by silica column chromatography (4-10% MeOH in $CH_2Cl_2$) yielding the desired product as an oil (23.9 mg, 0.0212 mmol, 35 % in two steps).

**$^1$H NMR** (400 MHz, $CD_2Cl_2$) $\delta_H$ (ppm) 8.38 (s, 1H), 7.87 (s, 1H), 7.46-6.85 (m, 18H), 5.46-5.21 (m, 4H), 5.19-4.73 (m, 3H), 4.30 (dd, *J* = 12.4, 4.9 Hz, 1H), 4.08 (d, *J* = 12.1 Hz, 1H), 3.91 (s, 1H), 2.41 (s, 4H), 2.02 (m, 12H), 1.26 (s, 31H), 0.92-0.83 (m, 3H).

**HR-MS** (ESI): $[C_{62}H_{80}N_2O_{15}S + H]^+$ calcd. 1125.5352, found 1125.5397. $[C_{62}H_{80}N_2O_{15}S + Na]^+$ calcd. 1147.5171, found 1147.5234. $[C_{62}H_{80}N_2O_{15}S + K]^+$ calcd. 1163.4911, found 1163.4950.

***R*f** (8 % MeOH in $CH_2Cl_2$) 0.53.

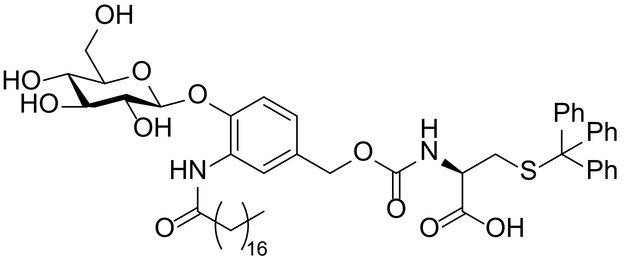


**11**

### 3.1.19 Synthesis of 11

**S4** (16.9 mg, 0.0150 mmol, 1.0 equiv.) was dissolved in anhydrous MeOH (1.5 mL) under an atmosphere of argon. A few drops of a 25 wt% solution of NaOMe in MeOH was added until the reaction mixture was strongly alkaline (pH > 10). After stirring for 18 hours the reaction mixture was neutralized with 1M HCl (aq.), concentrated *in vacuo* and purified directly by flash column chromatography (10 % MeOH in EtOAc → 1:1:1:7 MeCN/$H_2O$/MeOH/EtOAc) to yield trityl protected receptor **11** (10.6 mg, 0.0111 mmol, 74 %) as a hard oil.

**$^1$H NMR** (400 MHz, DMSO-$d_6$) $\delta_H$ (ppm) 9.11 (s, 1H), 8.17 (s, 1H), 7.39-7.10 (m, 16H), 7.01 (dd, *J* = 8.4, 2.1 Hz, 1H), 6.85 (s, 1H), 5.93 (s, 1H), 5.30 (s, 1H), 5.13 (s, 1H), 4.92 (s, 2H), 4.61 (d, *J* = 7.2 Hz, 2H), 3.78 (q, *J* = 6.8 Hz, 1H), 3.69 (d, *J* = 10.8 Hz, 1H), 3.55-3.45 (m, 2H), 3.17 (t, *J* = 9.0 Hz, 2H), 2.39 (dt, *J* = 31.4, 7.7 Hz, 4H), 1.56 (q, *J* = 7.3 Hz, 2H), 1.22 (s, 28H), 0.84 (t, *J* = 6.7 Hz, 3H).

**HR-MS** (ESI): $[C_{54}H_{72}N_2O_{11}S + Na]^+$ calcd. 979.4749, found 976.4749. $[C_{54}H_{72}N_2O_{11}S + K]^+$ calcd. 995.4489, found 995.4482.

***R*f** (1:1:1:7 MeCN/$H_2O$/MeOH/EtOAc) 0.47.

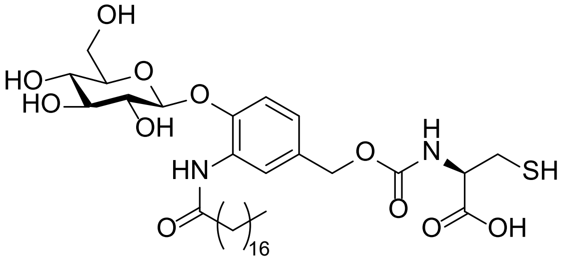


**12 (Glu-EAR)**

### 3.1.20 Synthesis of 12

According to **General synthesis method C**, **11** (7.3 mg, 0.0076 mmol, 1.0 equiv.) was reacted with (*i*-Pr)$_3$SiH (10 µL, 0.049 mmol, 6 equiv.) and trifluoroacetic acid (TFA) (20 µL, 0.26 mmol, 34 equiv.) in $CH_2Cl_2$ (0.5 mL). After reacting for 3.5 hours, the reaction mixture was concentrated in vacuo and purified directly by preparative HPLC (5% to 70% over 10 minutes, 70% to 90% over 25 minutes, 90% to 100% over 2 minutes and 100% to 5% over 2 minutes) to give **12 (Glu-EAR)** (2.0 mg, 0.0028 mmol, 37 %) as a white solid.

**HR-MS** (ESI): $[C_{35}H_{58}N_2O_{11}S + Na]^+$ calcd. 737.3653, found 737.3645.

$\boldsymbol{R_f}$ (1:1:1:7 MeCN/$H_2O$/MeOH/EtOAc) 0.38.

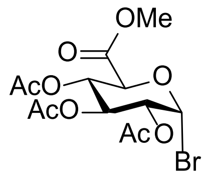


**S5**

### 3.1.21 Synthesis of S5

Methyl 1,2,3,4-tetra-*O*-acetyl-β-D-glucuronate (1022 mg, 2.716 mmol, 1.0 equiv.) was dissolved in $CH_2Cl_2$ (5 mL) under an atmosphere of argon and cooled to 0 °C. To the solution was added HBr (33 wt% in AcOH, 10 mL, 55 mmol, 20 equiv.) dropwise. The reaction mixture was allowed to reach r.t. After stirring for 4 hours, the mixture was poured onto ice and washed with ice water (x3) and $NaHCO_3$ (aq., sat., x3). The organic layer was dried over $MgSO_4$, filtered and concentrated *in vacuo* to give the acetobromoglucuronic acid methyl ester **S5** (1029 g, 2.591 mmol, 95%) as a sticky solid.

**$^1$H NMR** (400 MHz, $CDCl_3$) $\delta_H$ (ppm) 6.64 (d, *J* = 4.1 Hz, 1H), 5.61 (t, *J* = 9.7 Hz, 1H), 5.24 (dd, *J* = 10.3, 9.5 Hz, 1H), 4.85 (dd, *J* = 10.0, 4.1 Hz, 1H), 4.58 (d, *J* = 9.6 Hz, 1H), 3.76 (s, 3H), 2.10 (s, 3H), 2.05 (2 s, 6H).

**$^{13}$C NMR** (101 MHz, $CDCl_3$) $\delta_C$ (ppm) 169.8, 169.8, 169.6, 166.8, 85.5, 72.2, 70.5, 69.4, 68.6, 53.3, 20.8, 20.6.

**HRMS** (ESI): $[C_{13}H_{17}BrO_9 + Na]^+$ calcd. 418.9949, found 418.9954.

***R*f** (EtOAc/Pentane 2:3) 0.55.

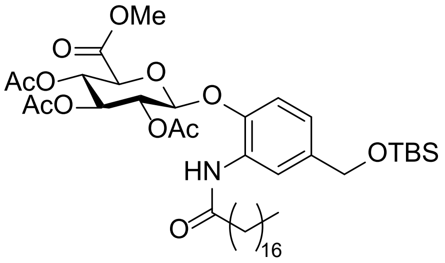


**13**

### 3.1.22 Synthesis of 13

A flask was evacuated during heating for 5 minutes and afterwards filled with an atmosphere of argon. Acetobromoglucuronic acid methyl ester **S5** (546.6 mg, 1.376 mmol, 1.0 equiv.), NaI (231.7 mg, 1.546 mmol, 1.1 equiv.) and $Na_2SO_4$ (dried at 120 °C, 542.1 mg) were mixed in anhydrous $CH_3CN$ (15 mL) and stirred for half an hour. The scaffold compound **3** (937.7 mg, 1.794 mmol, 1.3 equiv.) dissolved in anhydrous $CH_2Cl_2$ (25 mL) was added to the mixture. After additional half an hour, $Ag_2O$ (638.7 mg, 2.757 mmol, 2.0 equiv.) was added and the reaction mixture was left stirring at 30 °C in the dark for a total of 6 days. The reaction mixture was filtered over a plug of Celite® followed by washing with $CH_2Cl_2$. The filtrate was isolated and after removal of the solvent the crude was purified by flash column chromatography (EtOAc/pentane 1:5 → 1:4) to give the β-glucuronide **13** (47.2 mg, 0.0565 mmol, 4%) as a white solid.

**$^1$H NMR** (400 MHz, $CDCl_3$) $\delta_H$ (ppm) 8.33 (d, $J$ = 2.0 Hz, 1H), 7.87 (s, 1H), 7.05 (dd, $J$ = 8.4, 2.1 Hz, 1H), 6.90 (d, $J$ = 8.4 Hz, 1H), 5.45 – 5.25 (m, 3H), 5.04 (d, $J$ = 7.5 Hz, 1H), 4.67 (s, 2H), 4.17 (d, $J$ = 9.6 Hz, 1H), 3.75 (s, 3H), 2.41 (td, $J$ = 7.4, 3.1 Hz, 2H), 2.10 – 2.04 (m, 9H), 1.71 (m, 2H), 1.25 (m, $J$ = 6.3 Hz, 28H), 0.93 (s, 9H), 0.91 – 0.84 (m, 3H), 0.14 – 0.05 (m, 6H).

**$^{13}$C NMR** (101 MHz, $CDCl_3$) $\delta_C$ (ppm) 172.1, 170.2, 170.0, 169.5, 166.7, 144.1, 137.8, 129.2, 121.2, 118.4, 114.7, 100.5, 72.7, 71.3, 71.2, 69.4, 64.8, 53.2, 37.9, 32.1, 29.8, 29.8, 29.7, 29.6, 29.5, 26.1, 25.8, 22.8, 20.9, 20.7, 20.6, 18.6, 14.3, -5.1.

**HRMS** (ESI): $[C_4H_{73}NO_{12}Si + H]^+$ calcd. 836.4975, found 836.5012.

***R*f** (EtOAc/Pentane 1:4) 0.42.

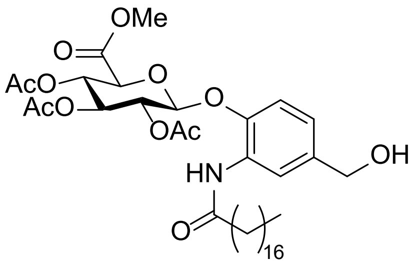

**S6**

### 3.1.23 Synthesis of S6

According to **General synthesis method A**, **13** (47.2 mg, 0.0565 mmol, 1.0 equiv.) and Conc. hydrochloric acid (conc., 0.05 mL, 0.6 mmol, 11 equiv.) in ethanol (1.5 mL) was reacted to produce **S6**, which was obtained by flash column chromatography (1:1 EtOAc/Pentane) to yield the benzyl alcohol as a colourless oil (19.0 mg, 0.0263 mmol, 47%).

**$^{1}$H NMR** (400 MHz, $CDCl_3$) $\delta_H$ (ppm) 8.39 (d, $J$ = 2.1 Hz, 1H), 7.89 (s, 1H), 7.04 (dd, $J$ = 8.4, 2.1 Hz, 1H), 6.92 (d, $J$ = 8.4 Hz, 1H), 5.44 – 5.25 (m, 3H), 5.06 (d, $J$ = 7.6 Hz, 1H), 4.61 (s, 2H), 4.19 (d, $J$ = 9.7 Hz, 1H), 3.75 (s, 3H), 2.41 (td, $J$ = 7.4, 3.3 Hz, 2H), 2.10 – 2.03 (3 s, 9H), 1.77 – 1.65 (m, 2H), 1.25 (m, 28H), 0.87 (t, $J$ = 6.8 Hz, 3H).

**$^{13}$C NMR** (101 MHz, $CDCl_3$) $\delta_C$ (ppm) 172.3, 170.2, 170.0, 169.6, 166.7, 144.5, 137.3, 129.4, 122.3, 119.5, 114.9, 100.3, 72.7, 71.2, 71.2, 69.3, 65.1, 53.3, 37.8, 32.1, 29.8, 29.8, 29.7, 29.6, 29.5, 29.5, 25.8, 22.8, 20.9, 20.7, 20.6, 14.3.

**HRMS** (ESI): $[C_{38}H_{59}NO_{12} + H]^+$ calcd. 722.4111, found 722.4131.

***R*f** (EtOAc/Pentane 1:1) 0.36.

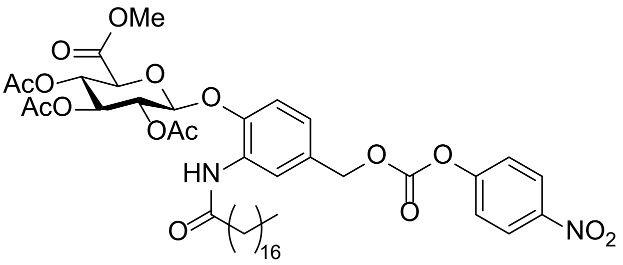


**S7**

### 3.1.24 Synthesis of S7

The linker with a free alcohol **S6** (19.0 mg, 0.0263 mmol, 1.0 equiv.) and $Et_3N$ (0.05 mL, 0.36 mmol, 14 equiv.) were dissolved in anhydrous $CH_2Cl_2$ (0.5 mL) under an atmosphere of argon. The mixture was cooled to 0 °C followed by dropwise addition of 4-nitrophenyl chloroformate (10.3 mg, 0.0511 mmol, 1.9 equiv.) in dry $CH_2Cl_2$ (0.5 mL). After stirring for 21 hours the reaction mixture was diluted with $CH_2Cl_2$ and washed with brine (x3). The organic layer was dried over $Na_2SO_4$, filtered and concentrated *in vacuo* to yield a yellow oil containing the carbonate **S7**. The crude was used directly in the following reaction without further purification.

***R*f** (EtOAc/Pentane 1:2) 0.70.

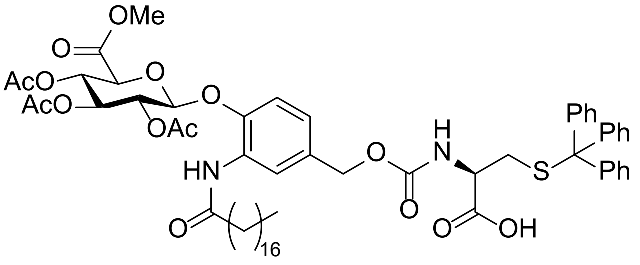


**S8**

### 3.1.25 Synthesis of S8

According to **General synthesis method B**, **S7** (23.7 mg, 0.0263 mmol, 1.0 equiv.) in dry $CH_2Cl_2$ (0.5 mL) was added to a stirring suspension of *S*-trityl-L-cysteine (18.1 mg, 0.0498 mmol, 1.9 equiv.) and triethylamine (TEA) (0.05 mL, 0.36 mmol, 14 equiv.) in dry $CH_2Cl_2$ (0.5 mL) and reacted overnight. Purification of **S8** was performed by silica column chromatography (4-10% MeOH in $CH_2Cl_2$) yielding the desired product as a white solid (14.5 mg, 0.0129 mmol, 49% in two steps).

**$^1$H NMR** (400 MHz, $CDCl_3$) $\delta_H$ (ppm) 8.28 (s, 1H), 7.85 (s, 1H), 7.46 – 7.18 (m, 5H), 7.16 – 6.76 (m, 12H), 5.52 – 5.13 (m, 4H), 5.08 – 4.48 (m, 3H), 4.35 – 3.75 (m, 2H), 3.71 – 3.46 (m, 3H), 2.75 – 2.15 (m, 4H), 1.99 (s, 9H), 1.35 – 1.09 (m, 28H), 0.80 (t, $J$ = 6.7 Hz, 3H).

**HRMS** (ESI): $[C_{61}H_{78}N_2O_{15}S + NH_4]^+$ calcd. 1128.5462, found 1128.5497.

***R*f** (6% MeOH in $CH_2Cl_2$) 0.27.

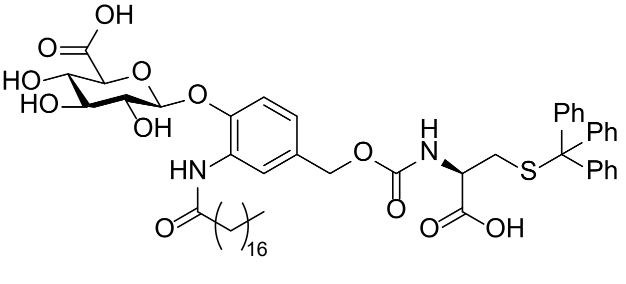

**14**

### 3.1.26 Synthesis of 14

Fully protected glucuronide **S8** (14.5 mg, 0.0129 mmol, 1.0 equiv.) was dissolved in anhydrous MeOH (0.5 mL) under an atmosphere of argon. A 25 wt% solution of NaOMe (in MeOH) was added dropwise until pH>10 and the reaction mixture was stirred for 19 hours. To the mixture were added $H_2O$ (1 mL) and 2 M NaOH (10 µL, 0.020 mmol, 1.6 equiv.) and the reaction mixture was left for another 10 minutes. The reaction mixture was neutralized with MeOH-washed amberlite 120H+ ion exchange resin followed by decanting and afterwards washing the beads with MeOH. The resulting solution was purified by preparative HPLC (10% to 100% for 20 minutes, 100% to 100% for 2 minutes and 100% to 10% for 2 minutes) and lyophilized to give the trityl protected GUS-EAR **14** (6.3 mg, 0.0065 mmol, 50%) as a white solid.

**$^1$H NMR** (400 MHz, DMSO) $\delta_H$ (ppm) 9.05 (s, 1H), 8.17 (d, $J$ = 2.0 Hz, 1H), 7.65 (d, $J$ = 8.5 Hz, 1H), 7.40 – 7.16 (m, 15H), 7.12 – 6.96 (m, 2H), 4.99 – 4.86 (m, 2H), 4.80 (d, $J$ = 7.4 Hz, 1H), 3.92 – 3.75 (m, 3H), 3.45 – 3.26 (m, 5H), 2.58 – 2.52 (m, 1H), 2.36 (m, 3H), 1.57 (t, $J$ = 7.3 Hz, 2H), 1.23 (s, 28H), 0.89 – 0.80 (m, 3H).

**HRMS** (ESI): $[C_{54}H_{69}N_2O_{12}S]^-$ calcd. 696.4576, found 696.4594.

***R*f** (EtOAc/MeOH/MeCN/$H_2O$ 7:1:1:1) 0.24.

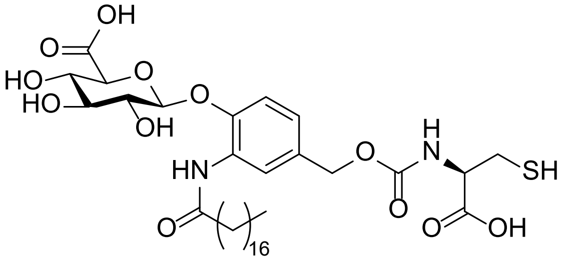


**15 (Gus-EAR)**

### 3.1.27 Synthesis of 15

According to **General synthesis method C**, **14** (3.1 mg, 0.0032 mmol, 1.0 equiv.) was reacted with (*i*-Pr)$_3$SiH (10 μL, 0.049 mmol, 15 equiv.) and trifluoroacetic acid (TFA) (20 μL, 0.26 mmol, 81 equiv.) in $CH_2Cl_2$ (1.0 mL). After reacting for 2 hours, the reaction mixture was purified by flash column chromatography (10% MeOH in $CH_2Cl_2$ → 7:1:1:1 EtOAc/MeOH/EtOH/$H_2O$) to yield GUS-EAR **15** (2.0 mg, 0.0027 mmol, 86%) as a white powder.

**HRMS** (ESI): $[C_{35}H_{55}N_2O_{12}S]^-$ calcd. 727.3481, found 727.3491.

***R*f** (EtOAc/MeOH/MeOH/$H_2O$ 7:1:1:1) 0.16.

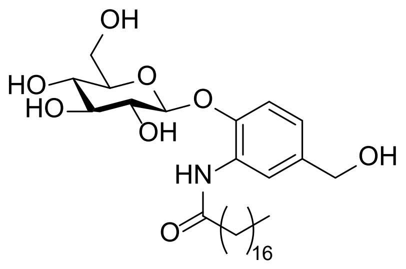

**S9**

### 3.1.28 Synthesis of S9

**S2** (37.9 mg, 0.052 mmol, 1.0 equiv.) was dissolved in anhydrous MeOH (3.4 mL) under an atmosphere of argon. The solution was made strongly alkaline (pH > 10) with a few drops of a 25% wt. solution of NaOMe in MeOH and left to stir at room temperature. After 4 days, the reaction mixture was neutralized with 1 M HCl (aq.), concentrated *in vacuo* and purified by flash column chromatography (5% MeOH/EtOAc) to afford the glucose-scaffold model receptor **S9** (17.6 mg, 0.031 mmol, 60%) as a white solid.

**$^{1}$H NMR** (400 MHz, DMSO-*d*$_6$): $\delta_H$ (ppm) 9.05 (s, 1H), 8.11 (s, 1H), 7.14 (d, *J* = 8.3 Hz, 1H), 6.94 (dd, *J* = 8.3, 2.1 Hz, 1H), 5.87 (s, 1H), 5.18 (s, 1H), 5.11 (t, *J* = 5.7 Hz, 1H), 5.06 (d, *J* = 5.1 Hz, 1H), 4.64 – 4.51 (m, 2H), 4.40 (d, *J* = 5.4 Hz, 2H), 3.70 (dd, *J* = 11.8, 5.5 Hz, 1H), 3.50 - 3.45 (m, 1H), 3.31 – 3.23 (m, 3H), 3.19 – 3.13 (m, 1H), 2.35 (t, *J* = 7.6 Hz, 2H), 1.57 (q, *J* = 7.2 Hz, 2H), 1.23 (s, 28H), 0.85 (t, *J* = 5.8 Hz, 3H).

**$^{13}$C NMR** (101 MHz, DMSO-*d*$_6$): $\delta_C$ (ppm) 171.2, 145.4, 137.4, 129.2, 121.6, 119.0, 117.7, 103.2, 77.2, 76.0, 73.3, 69.8, 62.8, 60.8, 36.4, 31.3, 29.1, 29.0, 28.9, 28.7, 28.7, 25.2, 22.1, 14.0.

**HRMS** (ESI+): $[C_{31}H_{53}NO_8Na]^+$: calcd. 590.3663, found: 590.3665; calcd. for $[C_{31}H_{54}NO_8]^+$: 568.3844, found: 568.3855.

***R*f** (10% MeOH/EtOAc) 0.49.

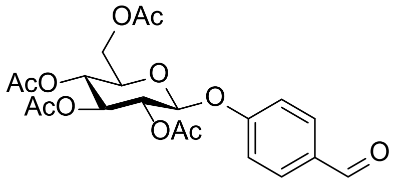

**S10**

### 3.1.29 Synthesis of S10

The protected α-glucosyl bromide **S1** (530.5 mg, 1.29 mmol, 1.0 equiv.), 4-hydroxybenzaldehyde (315.0 mg, 2.58 mmol, 2.0 equiv.) and 3 A mol sieves (550 mg) were added to a Schenk-dried flask and dissolved in anhydrous MeCN (32 mL). The suspension was left to stir for 30 min at room temperature under an atmosphere of argon before $Ag_2O$ (747.0 mg, 3.23 mmol, 2.5 equiv.) was added. The reaction mixture was left to stir in the dark for 22 h. The reaction mixture was then filtered over a plug of Celite® and washed with $CH_2Cl_2$. The filtrate was concentrated *in vacuo* and the crude was purified by flush column chromatography (gradient, 25% to 40% EtOAc/pentane) yielding the desired benzylic aldehyde **S10** as white crystals (396.0 mg, 0.88 mmol, 68%).

**$^1$H NMR** (400 MHz, $CDCl_3$): $\delta_H$ (ppm) 9.92 (s, 1H), 7.84 (d, *J* = 8.9 Hz, 2H), 7.09 (d, *J* = 8.7 Hz, 2H), 5.35 – 5.28 (m, 2H), 5.24 – 5.15 (m, 2H), 4.28 (dd, *J* = 12.3, 5.6 Hz, 1H), 4.17 (dd, *J* = 12.3, 2.4 Hz, 1H), 3.92 (ddd, *J* = 10.0, 5.4, 2.4 Hz, 1H), 2.06 – 2.04 (4s, 12H).

**$^{13}$C NMR** (101 MHz, $CDCl_3$): $\delta_C$ (ppm) 190.8, 170.6, 170.3, 169.5, 169.3, 161.3, 132.0, 131.9, 116.9, 98.1, 72.6, 72.4, 71.1, 68.2, 62.0, 20.8, 20.7, 20.7, 20.7.

**HRMS** (ESI+): $[C_{21}H_{24}O_{11}Na]^+$: calcd. 475.1211, found 475.1235; $[C_{21}H_{26}O_{11}K]^+$: calcd. 491.0951, found 491.0971.

***$R_f$*** (50% EtOAc/pentane) 0.43.

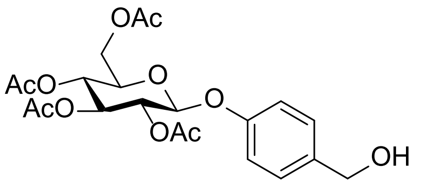

**S11**

### 3.1.30 Synthesis of S11

In a flame dried flask the benzylic aldehyde **S10** (390.0 mg, 0.86 mmol, 1.0 equiv.) was dissolved in a mixture of $CHCl_3$/*i*-PrOH (7.6 mL/1.9 mL) and cooled to 0 °C under an atmosphere of argon. Silica gel (380 mg) was then added to the reaction mixture and the suspension was left to stir for 10 min before $NaBH_4$ (65.2 mg, 1.72 mmol, 2.0 equiv.) was added in one potion. After 2 h, additional $NaBH_4$ (20.0 mg, 0.53 mmol, 0.6 equiv.) was added and reaction mixture was left to stir for 30 min more. Upon completion, the reaction mixture was filtered over a plug of Celite® and washed with $CH_2Cl_2$. The filtrate was washed with brine (x 3), dried over $MgSO_4$, filtered and concentrated *in vacuo* to afford the benzylic alcohol **S11** as a white foam (379.8 mg, 0.836 mmol, 97%).

**$^1$H NMR** (400 MHz, $CDCl_3$): $\delta_H$ (ppm) 7.30 (d, $J$ = 8.6 Hz, 2H), 6.98 (d, $J$ = 8.6 Hz, 2H), 5.31 – 5.24 (m, 2H), 5.16 (t, $J$ = 10.0 Hz, 1H), 5.06 (d, $J$ = 7.4 Hz, 1H), 4.64 (s, 2H), 4.28 (dd, $J$ = 12.2, 5.3 Hz, 1H), 4.16 (dd, $J$ = 12.4, 2.4 Hz, 1H), 3.85 (ddd, $J$ = 10.0, 5.3, 2.5 Hz, 1H), 2.07 – 2.03 (4s, 12H).

**$^{13}$C NMR** (101 MHz, $CDCl_3$): $\delta_C$ (ppm) 170.7, 170.4, 169.6, 169.5, 156.5, 136.1, 128.6, 117.2, 99.3, 72.8, 72.2, 71.3, 68.4, 64.9, 62.1, 20.8, 20.8, 20.8, 20.7.

**HRMS** (ESI+): $[C_{21}H_{26}O_{11}Na]^+$: calcd. 477.1367, found 477.1390; $[C_{21}H_{26}O_{11}K]^+$: calcd. 493.1107, found 493.1104.

***R*f** (60% EtOAc/pentane) 0.35.

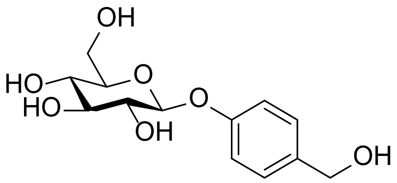

**S12**

### 3.1.31 Synthesis of S12

The protected glucoside **S11** (9.8 mg, 0.022 mmol, 1.0 equiv.) was dissolved in anhydrous MeOH (1 mL). To this was added a few drops of NaOMe (25 wt% in MeOH) until pH > 10. The reaction mixture was stirred for 18 hours and concentrated *in vacuo*. The residue was purified directly by preparative HPLC (5% to 15% for 15 minutes, 15% to 100% for 7 minutes, 100% to 100% for 2 minutes and 100% to 5% for 2 minutes) to give the glucoside **S12** (4.2 mg, 0.015 mmol, 67%) as a white solid.

**HRMS** (ESI+): $[C_{13}H_{18}O_7Na]^+$: calcd. 309.0945, found 309.0960; $[C_{13}H_{18}O_7K]^+$: calcd. 325.0685, found 325.0692.

## 3.2 Protocols for secondary messenger release studies (HPLC)

### 3.2.1 Phos-EAR

A stock solution of **8** in DMSO (10 mM) was diluted in HEPES buffer (50 mM, pH = 7.5) to a final concentration of 0.1 mM. The compound was treated with ALP (0.1 g/L), ceria nanoparticles (1 g/L) or no activator at 37 °C for 2 hours. The ALP-treated sample was filtered through a 10K spin-filter to remove the enzyme. The ceria nanoparticle-treated sample was diluted with MeCN was diluted in the ratio 1:1, centrifuged, and the supernatant was collected. A reference with trityl cysteine was prepared in a similar manner. All samples were analyzed by HPLC. The mobile was initially 10% MeCN and was gradually increased to 100% MeCN over 20 minutes. The eluent was then kept at 100% MeCN for 8 minutes until the analysis was stopped. UV signals were detected at wavelengths 220 nm and 254 nm. Results: $t_r$ (**8**): 14.78 min, $t_r$ (Trt-Cys): 13.92 min, $t_r$ (**8** + ALP): 13.95 min, $t_r$ (**8** + $CeO_2$ NP): 13.96 min.

### 3.2.2 Glu-EAR

A stock solution of **11** in DMSO (2.5 mM) was diluted in HEPES buffer (20 mM, pH = 6.8) to a final concentration of 0.1 mM. The compound was treated with β-glucosidase (GLU) (0.1 g/L) or no activator at 37 °C for 2 hours. A reference with trityl cysteine was prepared in a similar manner. All samples were injected directly into the HPLC system equipped with a guard column. The mobile phase started a 5% B and was gradually increased to 100% within 18 minutes. The B eluent was kept at 100% for another 10 minutes. UV signals were detected at wavelengths 220 nm and 254 nm. Results: $t_r$ (**11**): 25.12 min, $t_r$ (Trt-Cys): 13.74 min, $t_r$ (**11** + GLU): 25.11 min.

### 3.2.3 Gus-EAR

A solution of compound **14** was prepared in PBS buffer (2.5 mM, pH = 7.4) at a concentration of 0.1 mM. The compound was incubated for 2 hours at 37 °C either in the absence or the presence of β-glucuronidase (GUS) (0.1 g/L). A reference with trityl cysteine was prepared in a similar manner. The GUS-treated sample was spin-filtered through a 30K spin-filter to remove the enzyme and injected into the HPLC. All samples were analyzed by HPLC. The mobile phase started a 5% B and was gradually increased to 100% within 18 minutes. The B eluent was kept at 100% for another 10 minutes. UV signals were detected at wavelengths 220 nm and 254 nm. Results: $t_r$ (**14**): 24.55 min, $t_r$ (Trt-Cys): 13.04 min, $t_r$ (**14** + GUS): 12.73 min.

### 3.2.4 Effect of $C_{18}$ anchor on GLU mediated cleavage (supplemental figure 1)

A stock solution of **S12** or **S9** in DMSO (10 mM) was diluted in HEPES buffer (20 mM, pH = 6.8) to a final concentration of 1 mM. The compound was treated with GLU (0.1 g/L) or no activator at 37 °C for 2 hours. A reference with 4-HBA or scaffold was prepared in a similar manner. All samples were injected directly into the HPLC system equipped with a guard column. The mobile phase started a 5% B and was gradually increased to 100% within 18 minutes. The B eluent was kept at 100% for another 10 minutes. UV signals were detected at wavelengths 220 nm and 254 nm. Results: $t_r$ (**S12**): 5.19 min, $t_r$ (4-HBA): 7.16 min, $t_r$ (**S12** + GLU): 7.18 min and $t_r$ (**S9**): 19.61 min, $t_r$ (Scaffold): 14.17 min, $t_r$ (**S9** + GLU): 19.61 min.

## 3.3 NMR spectra

### 3.3.1 NMR compound 1

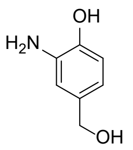


**1**

$^{1}H$ NMR (400 MHz)
$CD_3OD$

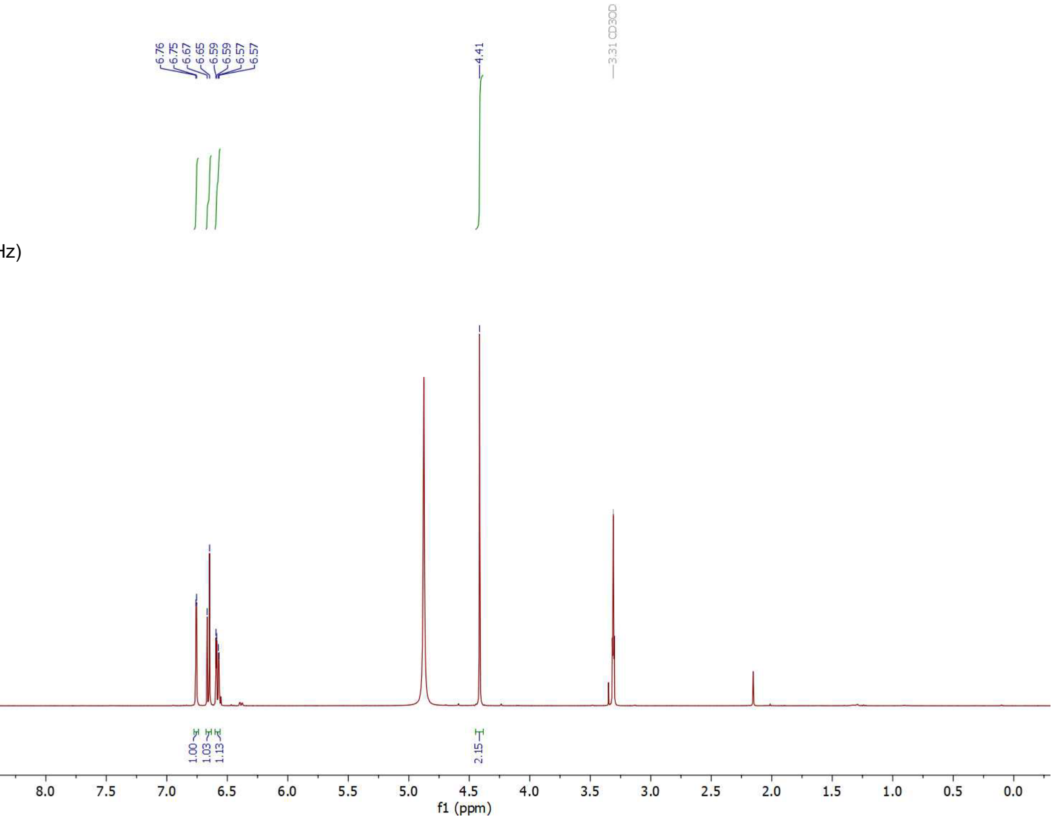


**1**

$^{13}C$ NMR (101 MHz)
$CD_3OD$

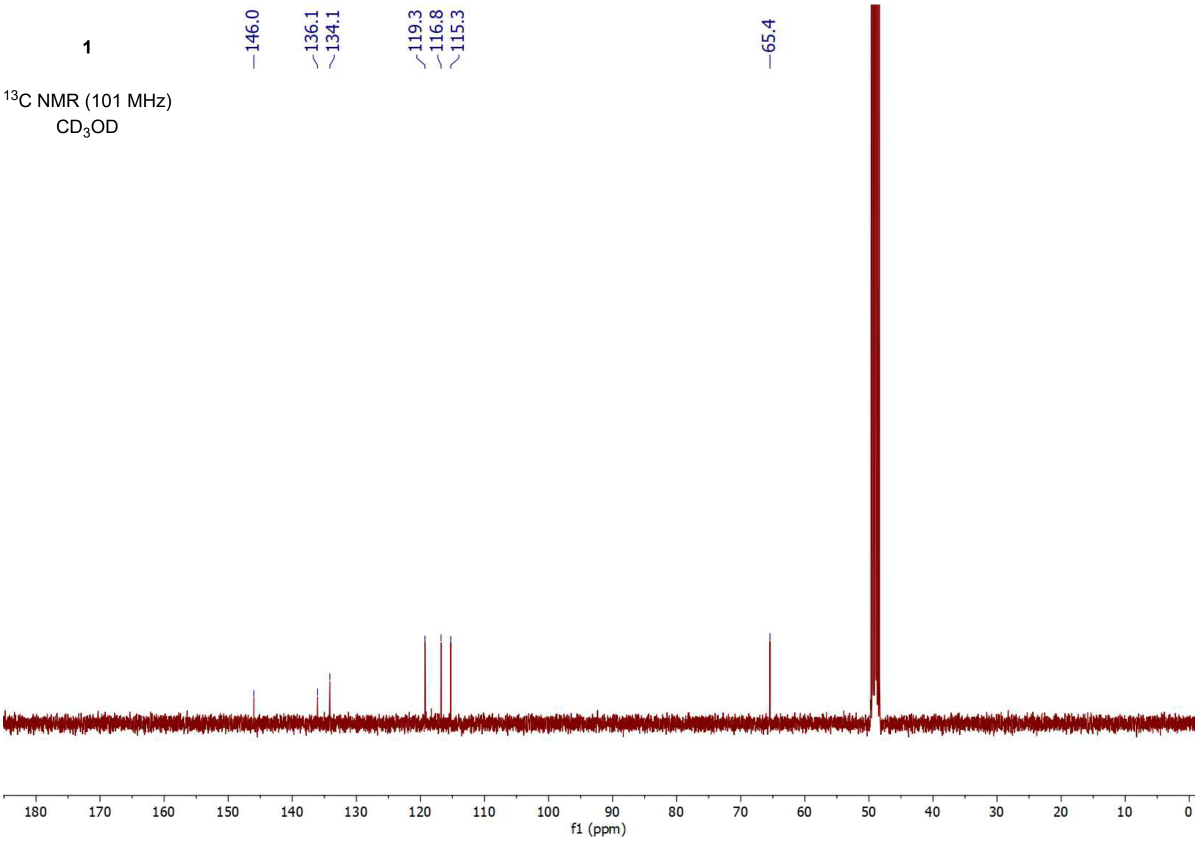

### 3.3.2 NMR compound 2

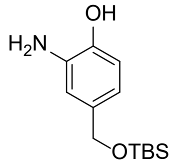


**2**

$^1H$ NMR (400 MHz)
$CDCl_3$

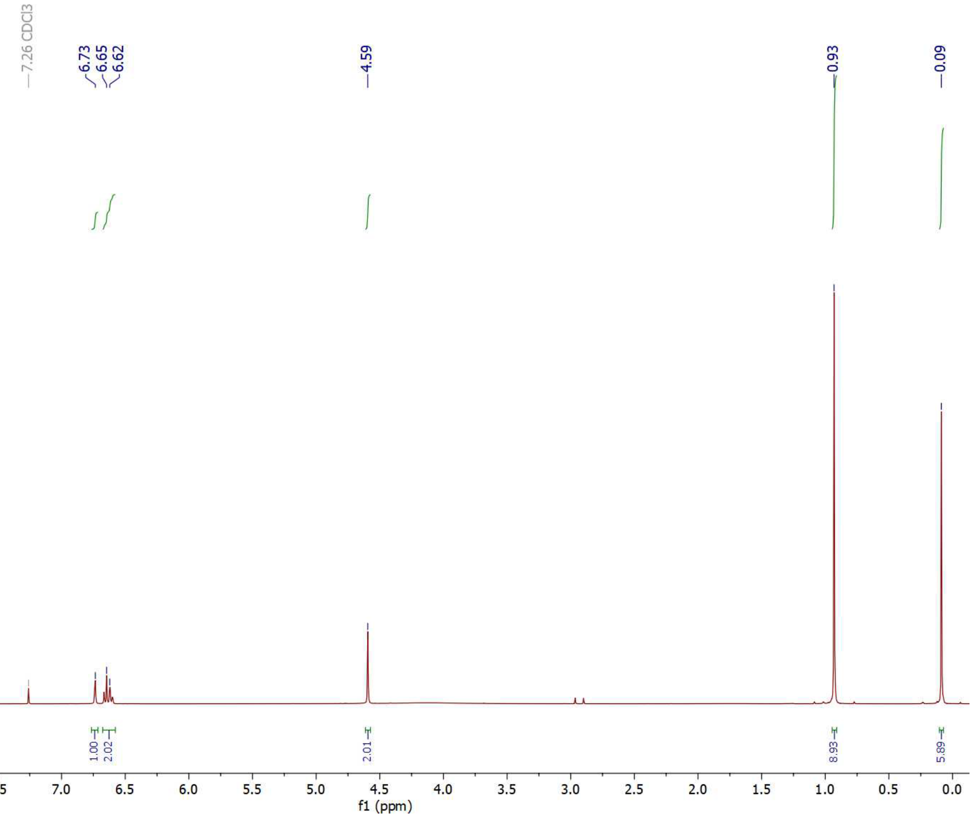


**2**

$^{13}C$ NMR (101 MHz)
$CDCl_3$

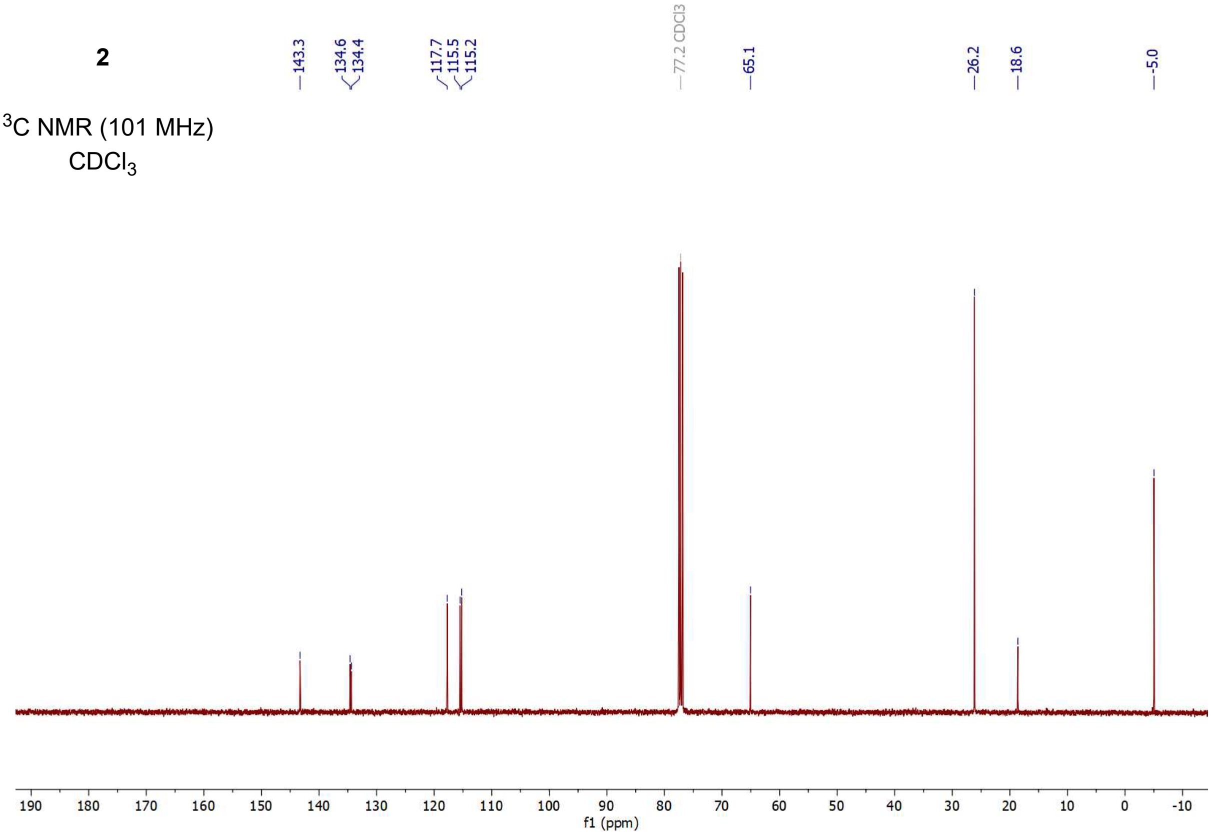

### 3.3.3 NMR compound 3

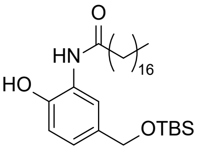


**3**

$^1$H NMR (400 MHz)

$CDCl_3$

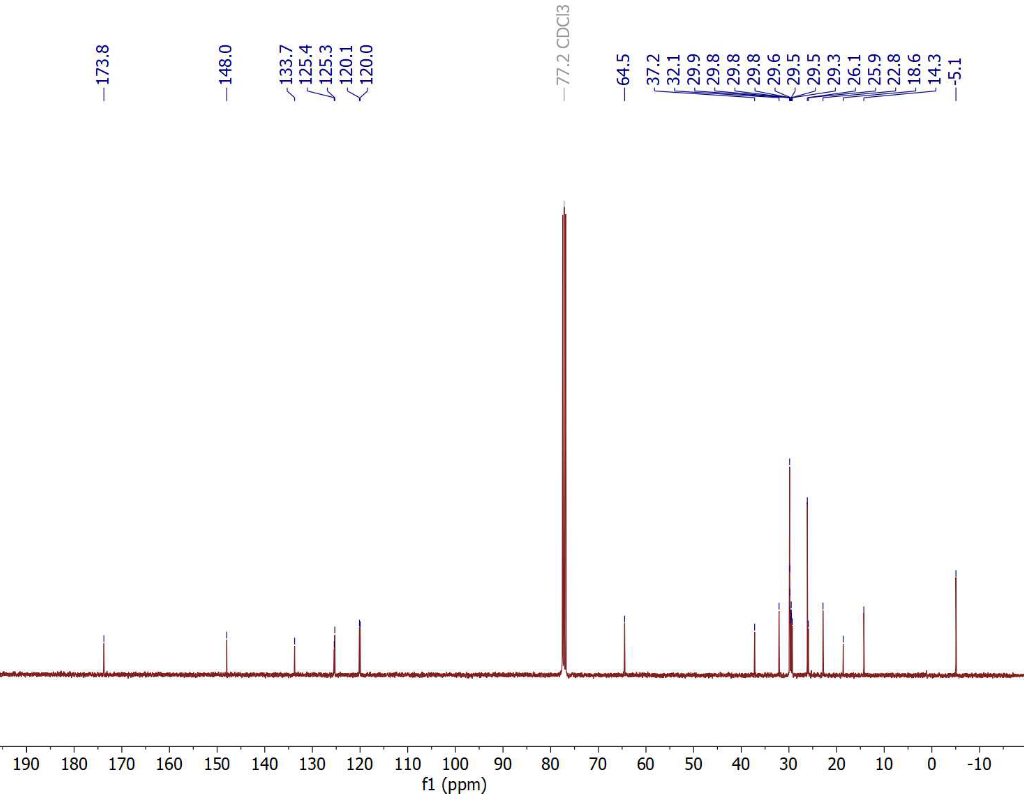


**3**

$^{13}$C NMR (101 MHz)

$CDCl_3$

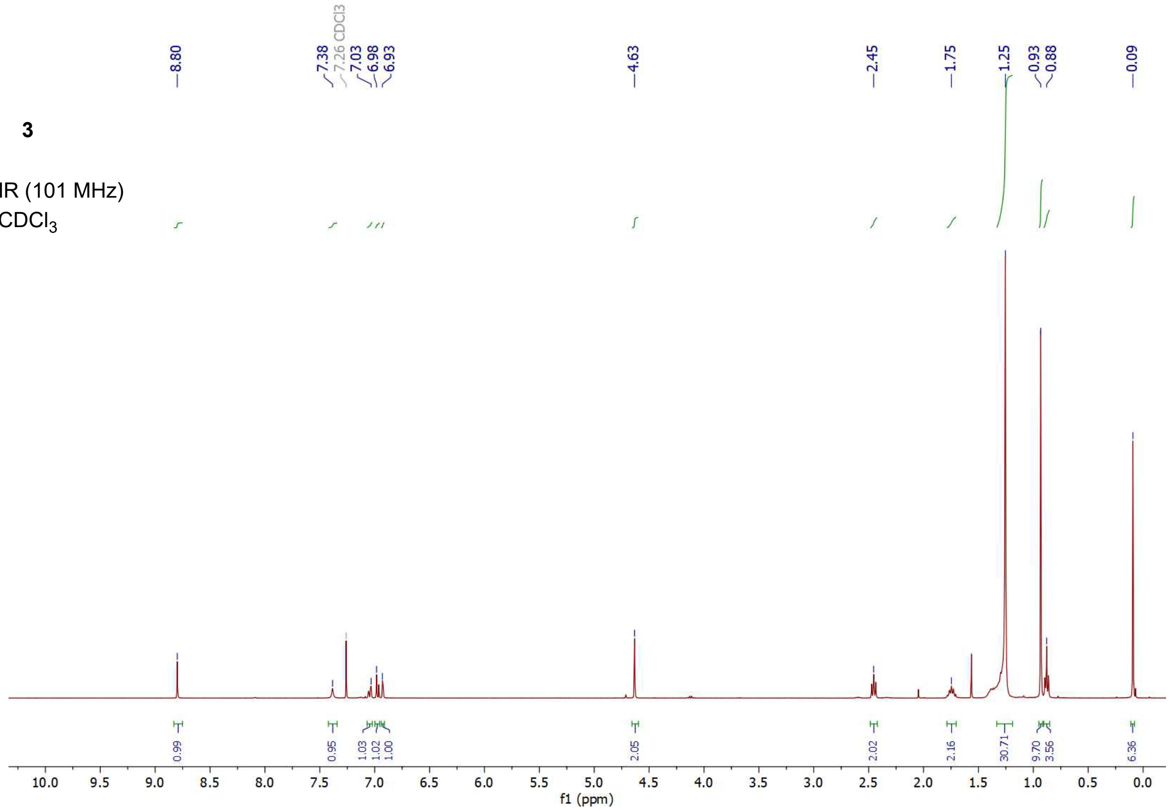

### 3.3.4 NMR compound All-4

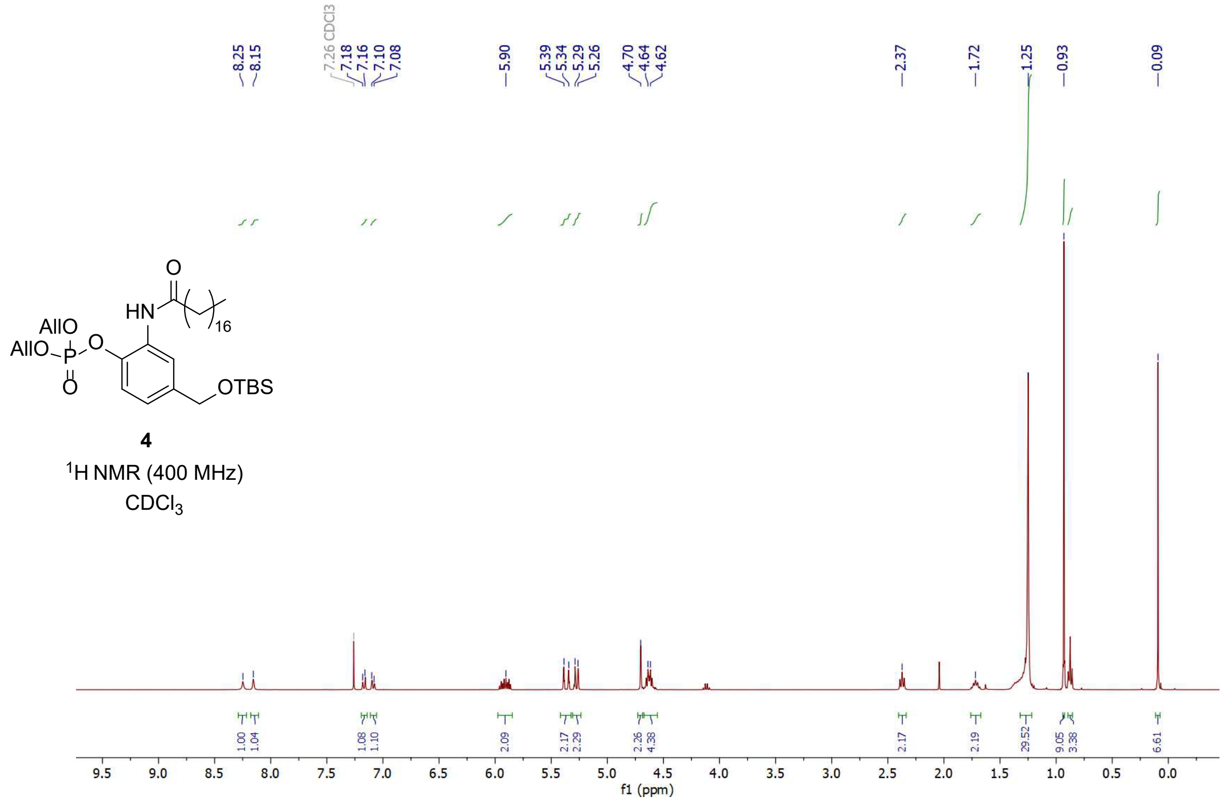


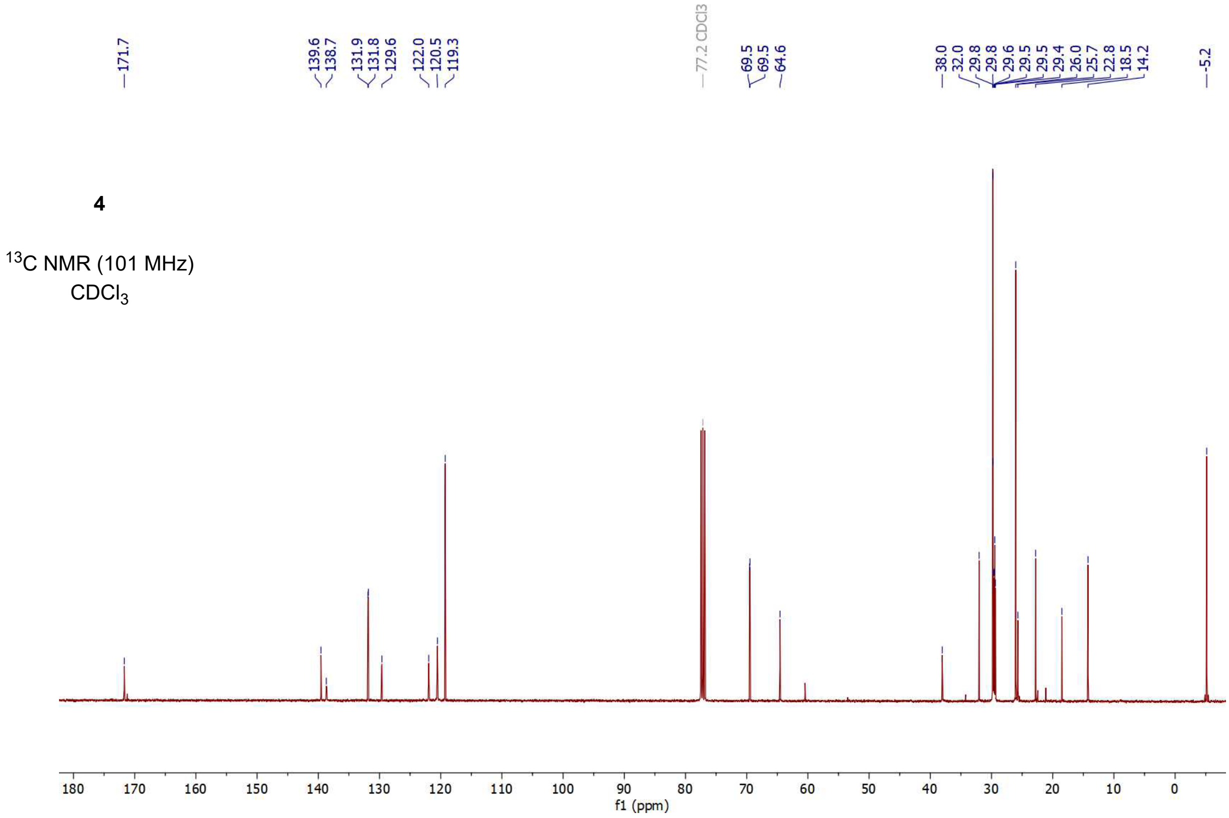

## 3.3.5 NMR compound All-5

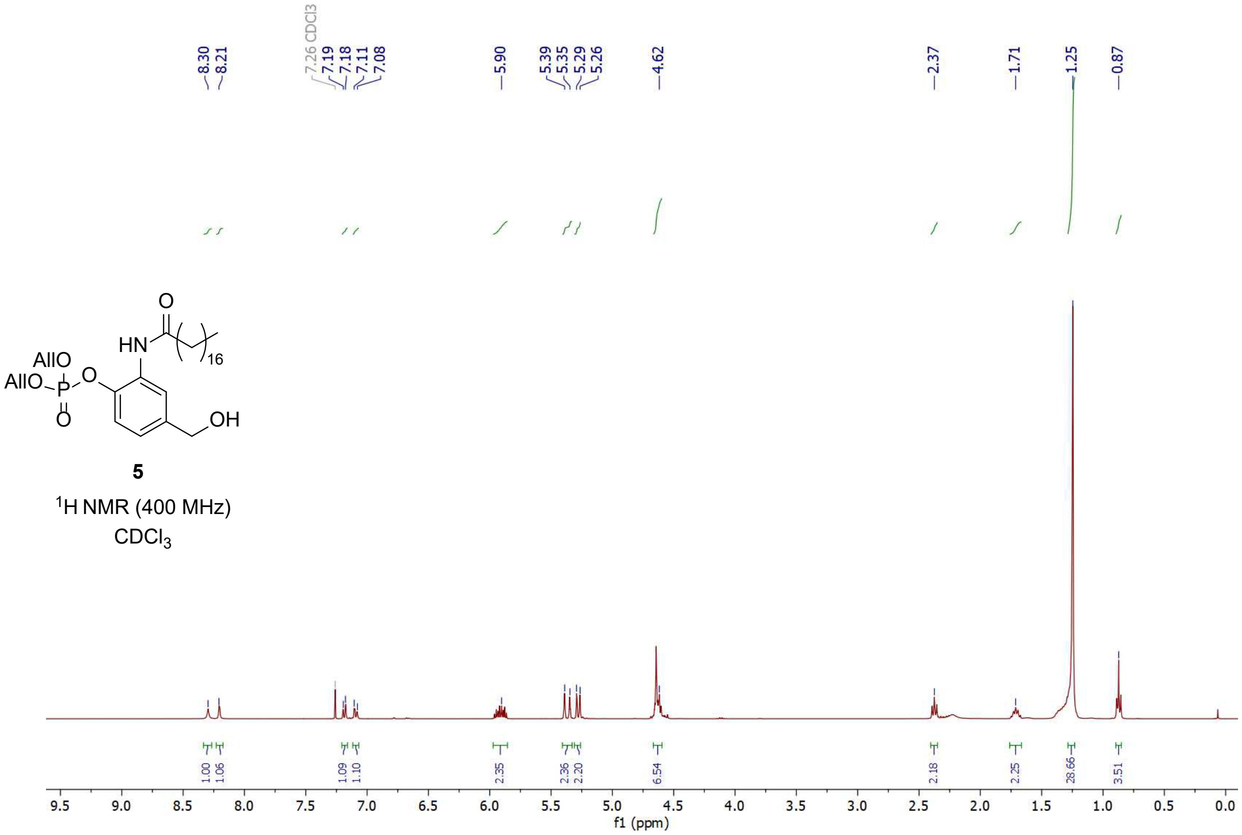


**5**

$^{1}$H NMR (400 MHz)
$CDCl_3$

**5**

$^{13}$C NMR (101 MHz)
$CDCl_3$

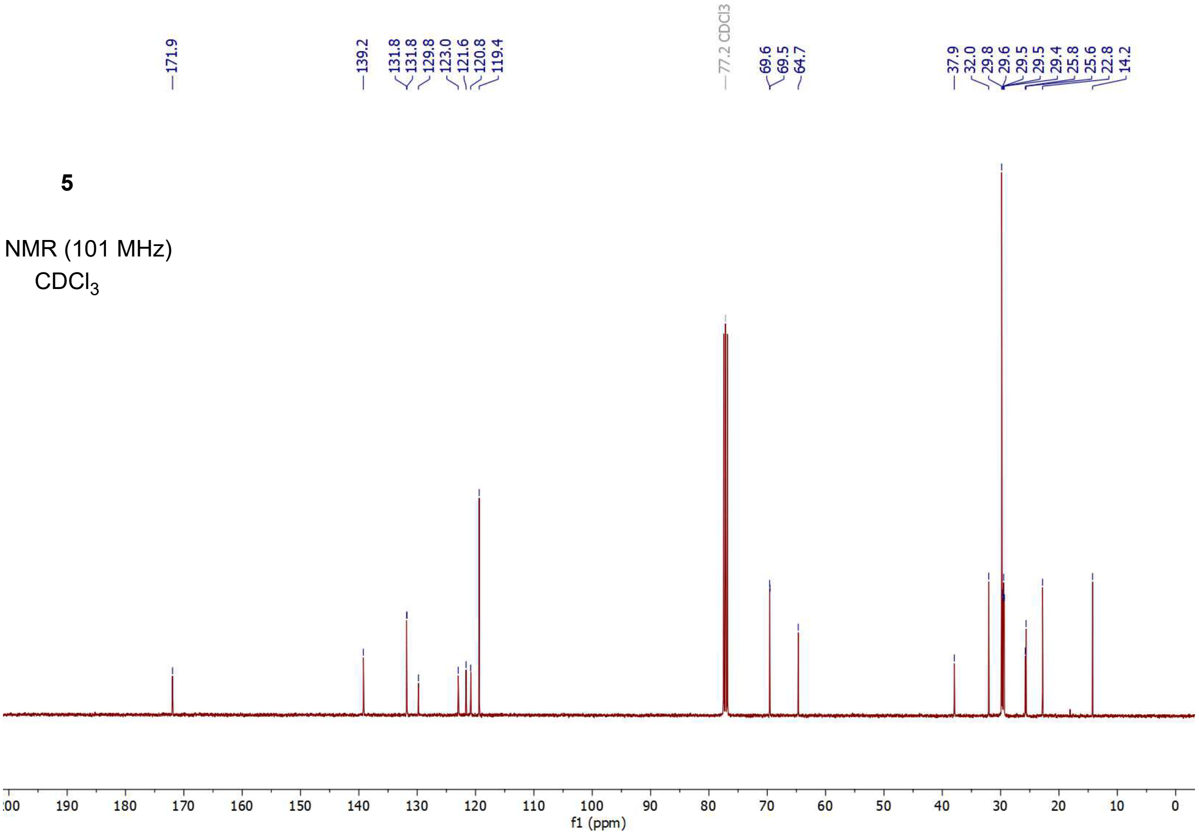

### 3.3.6 NMR compound All-6

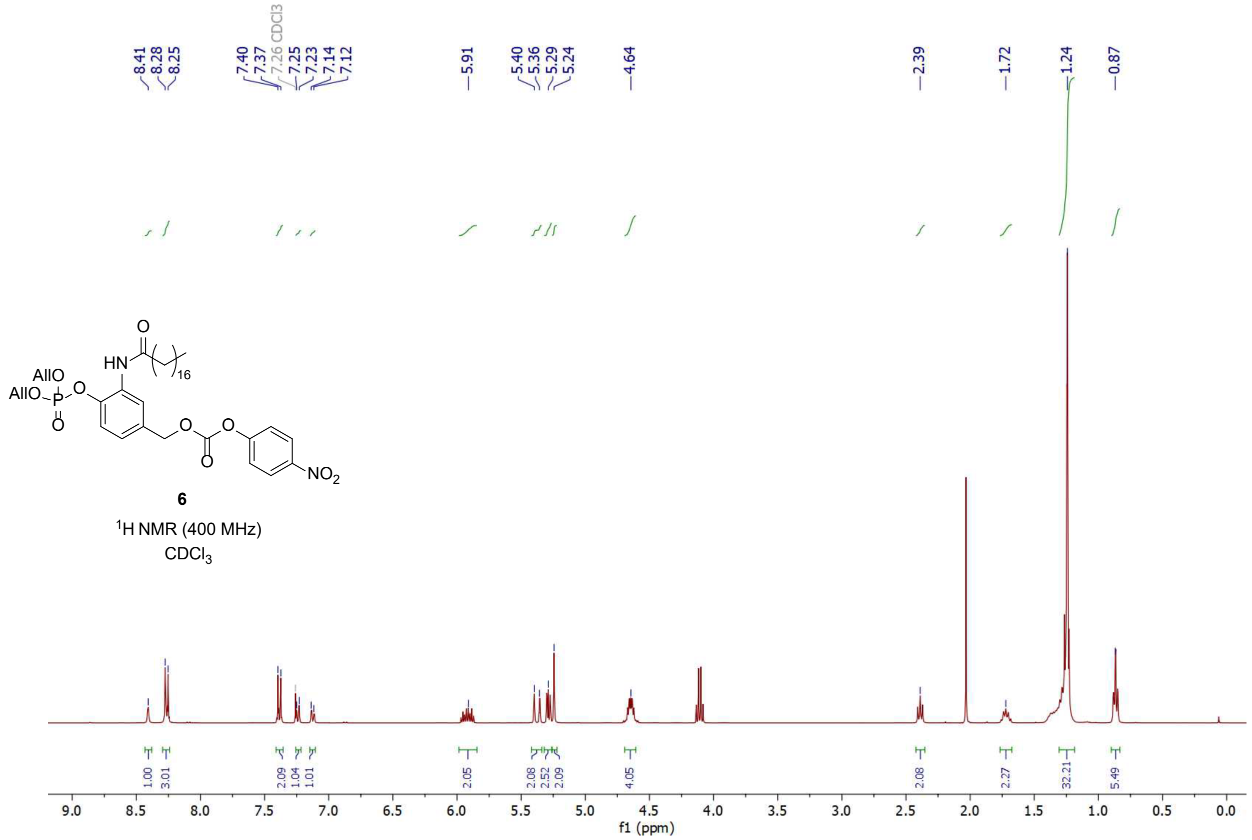


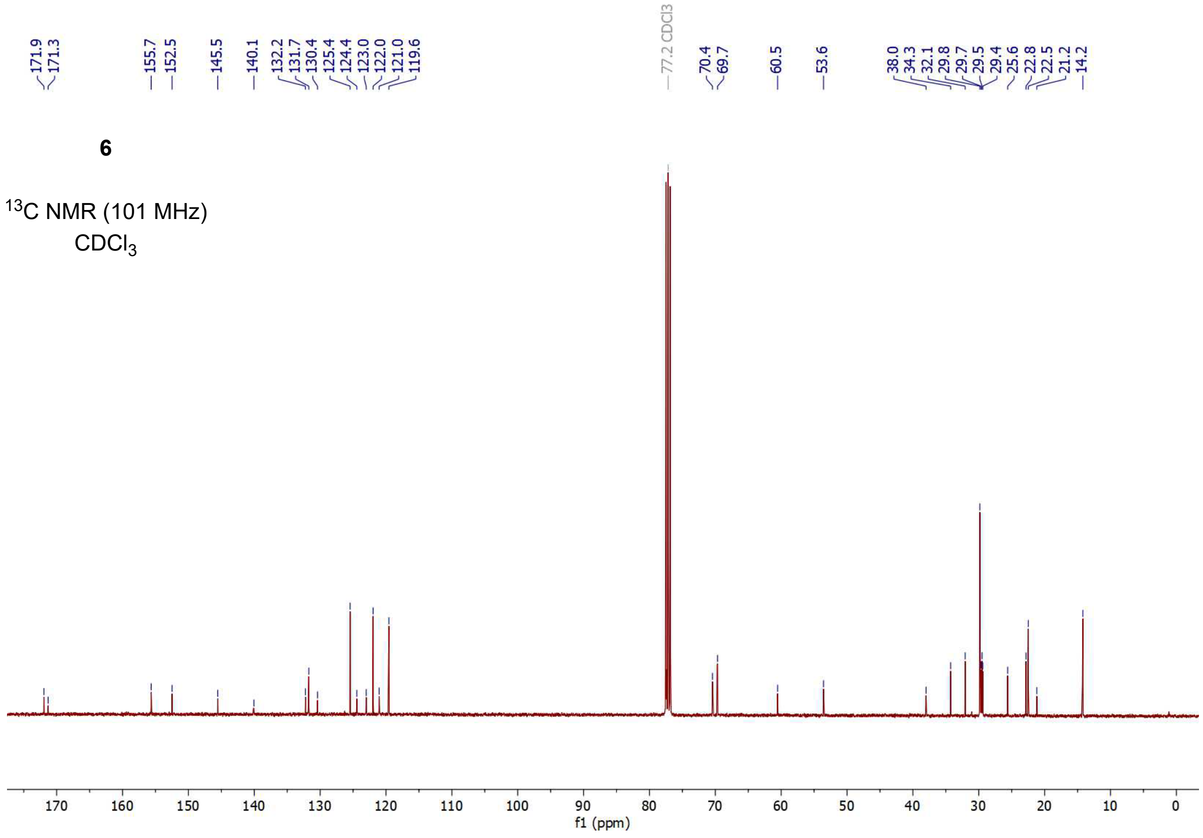

## 3.3.7 NMR compound All-7

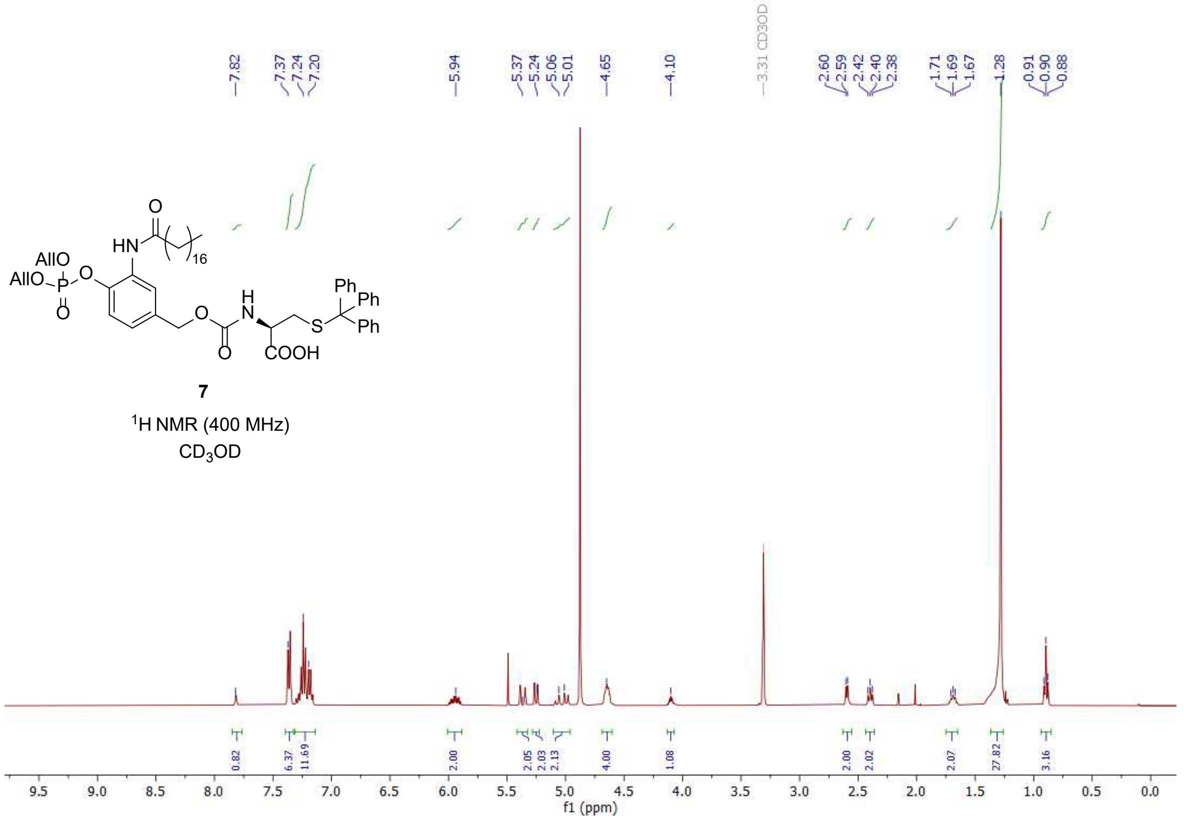


## 3.3.8 NMR compound 8

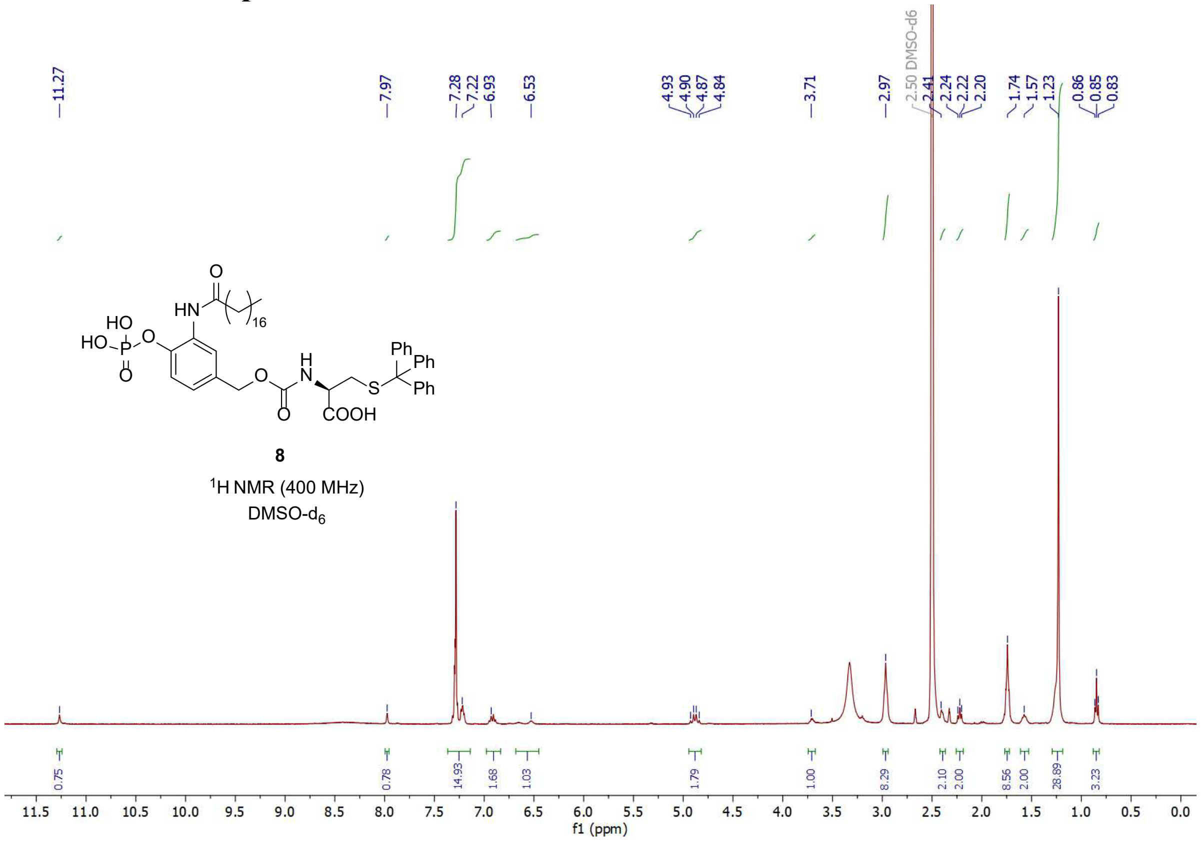

### 3.3.9 NMR compound S1

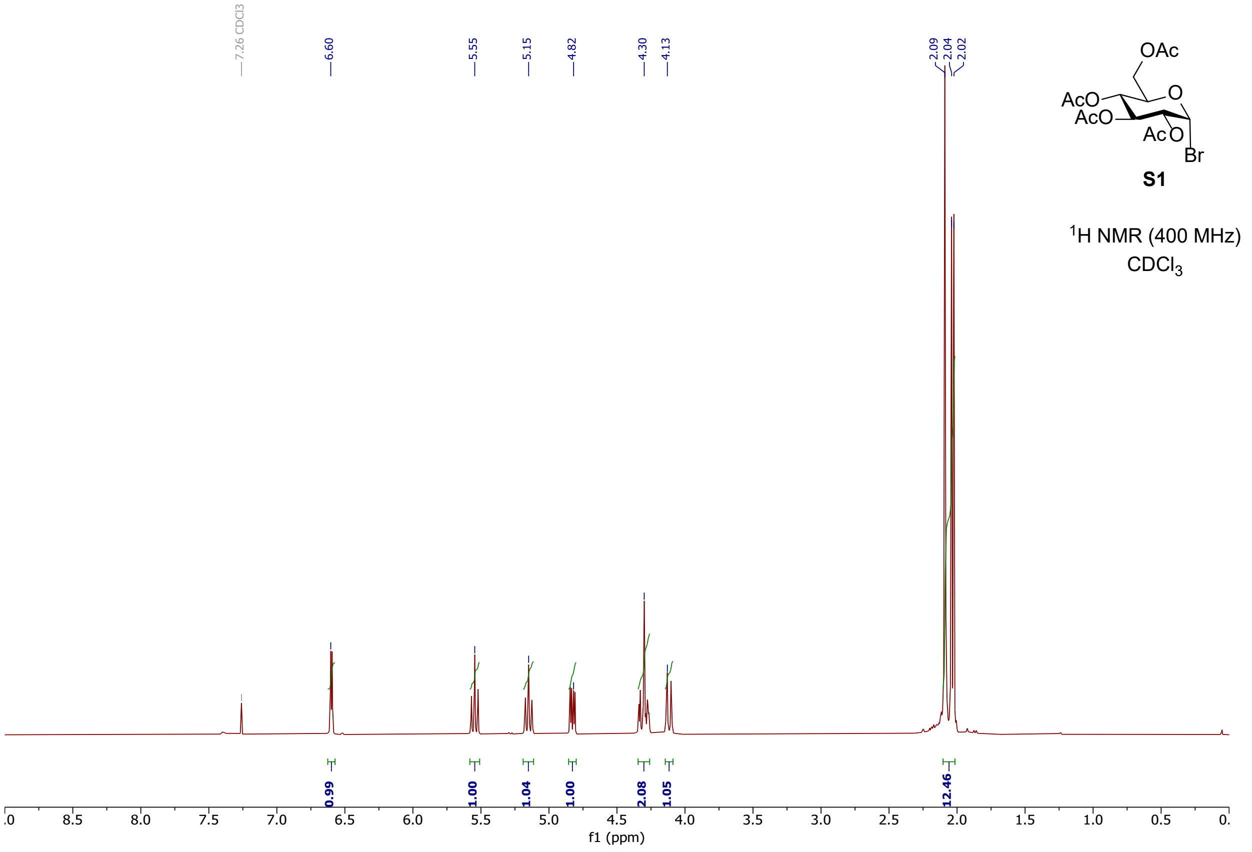


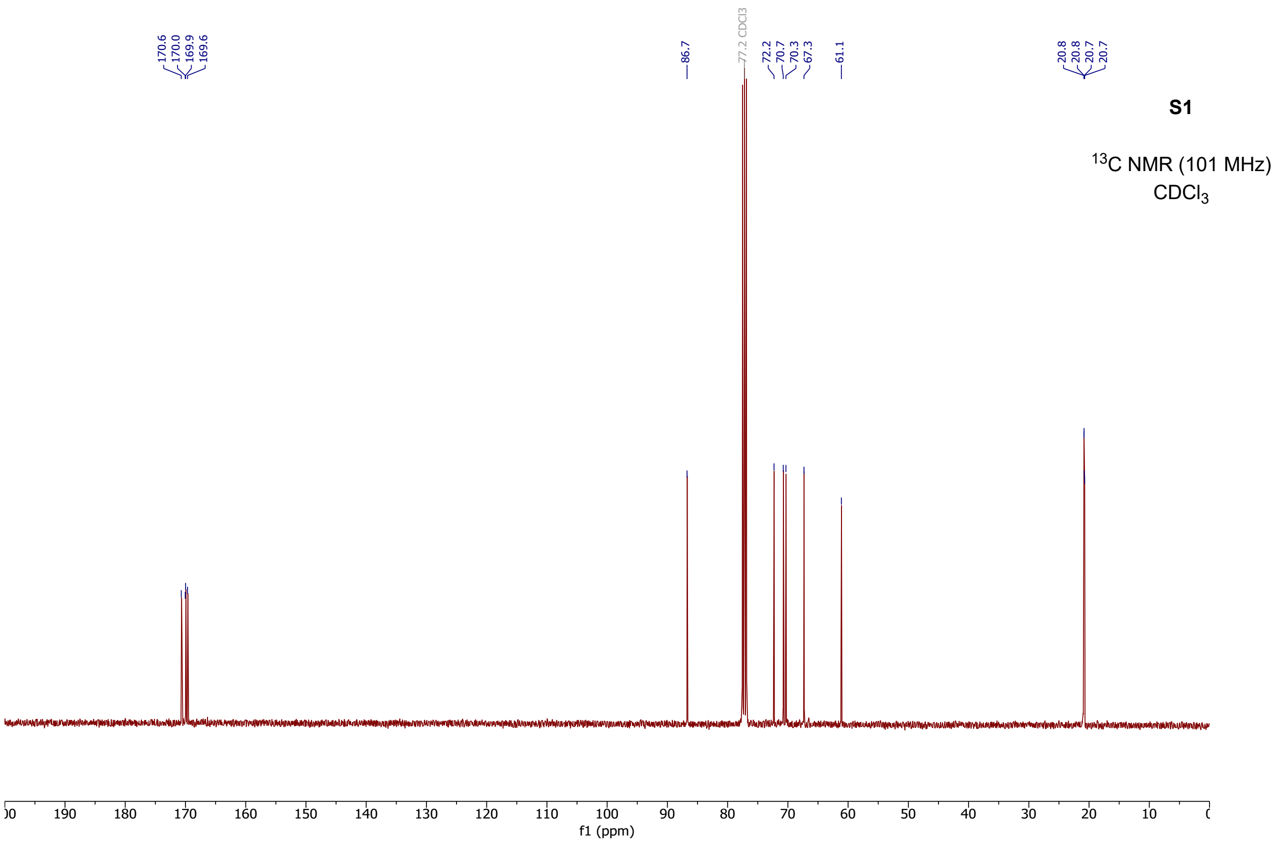

### 3.3.10 NMR compound 10

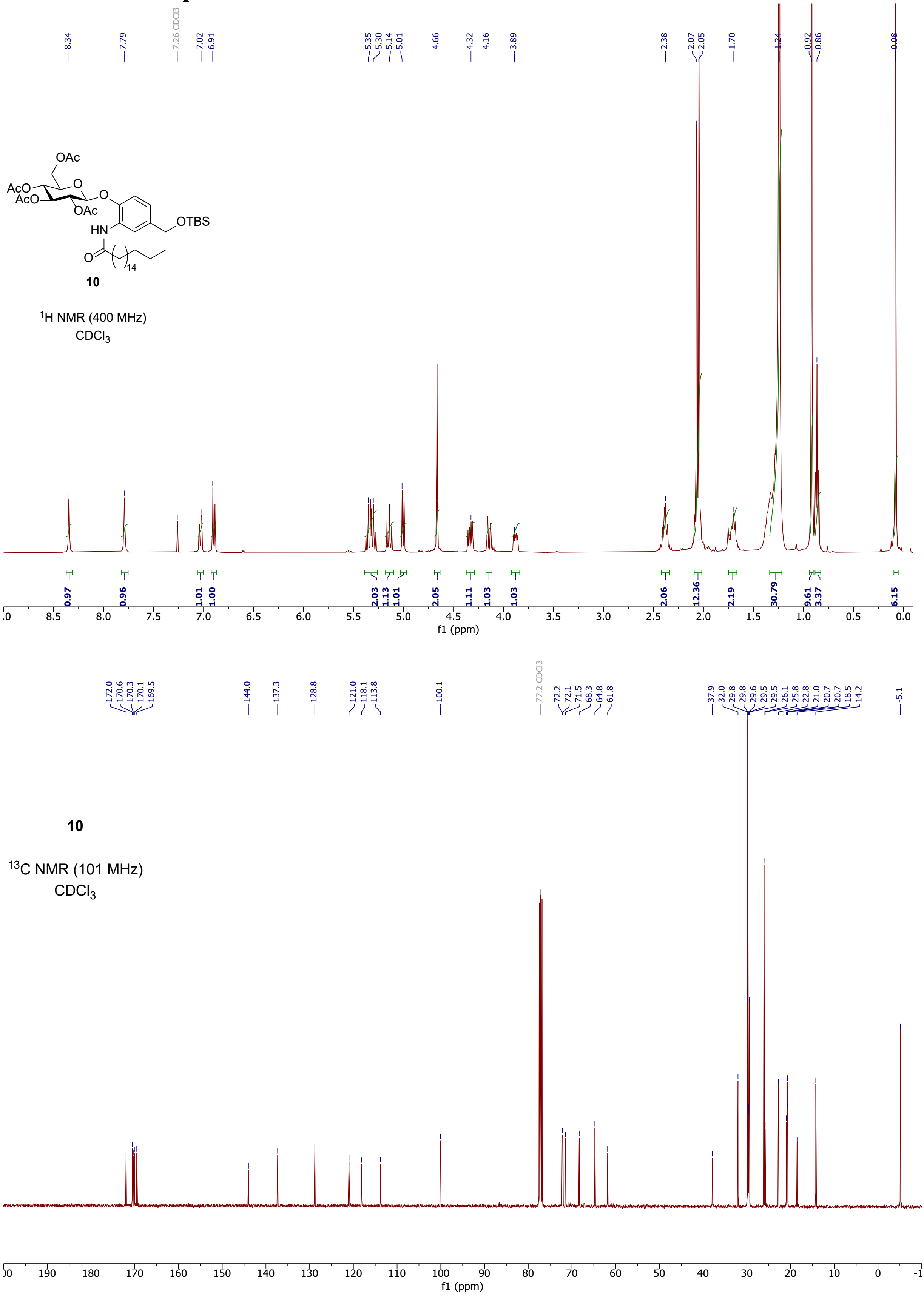

## 3.3.11 NMR compound S2

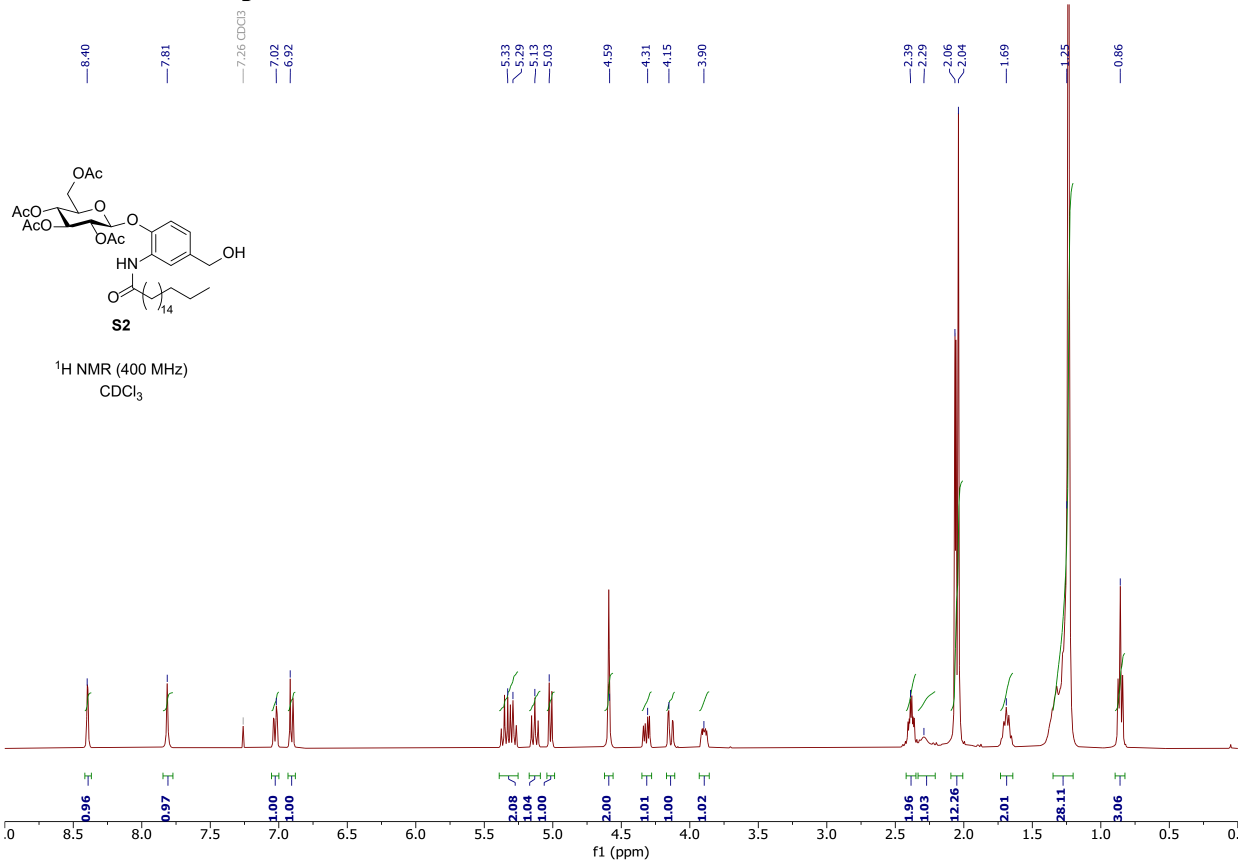


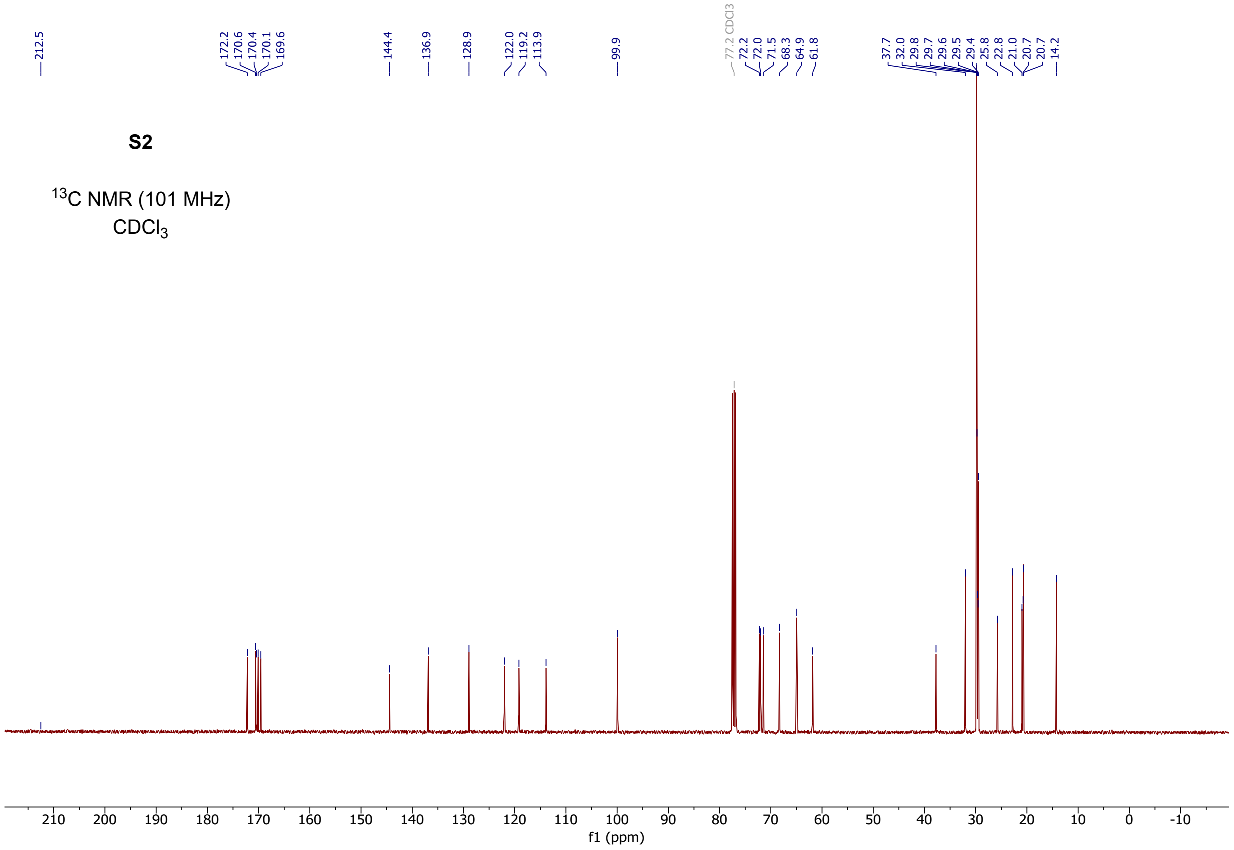

## 3.3.12 NMR compound S4

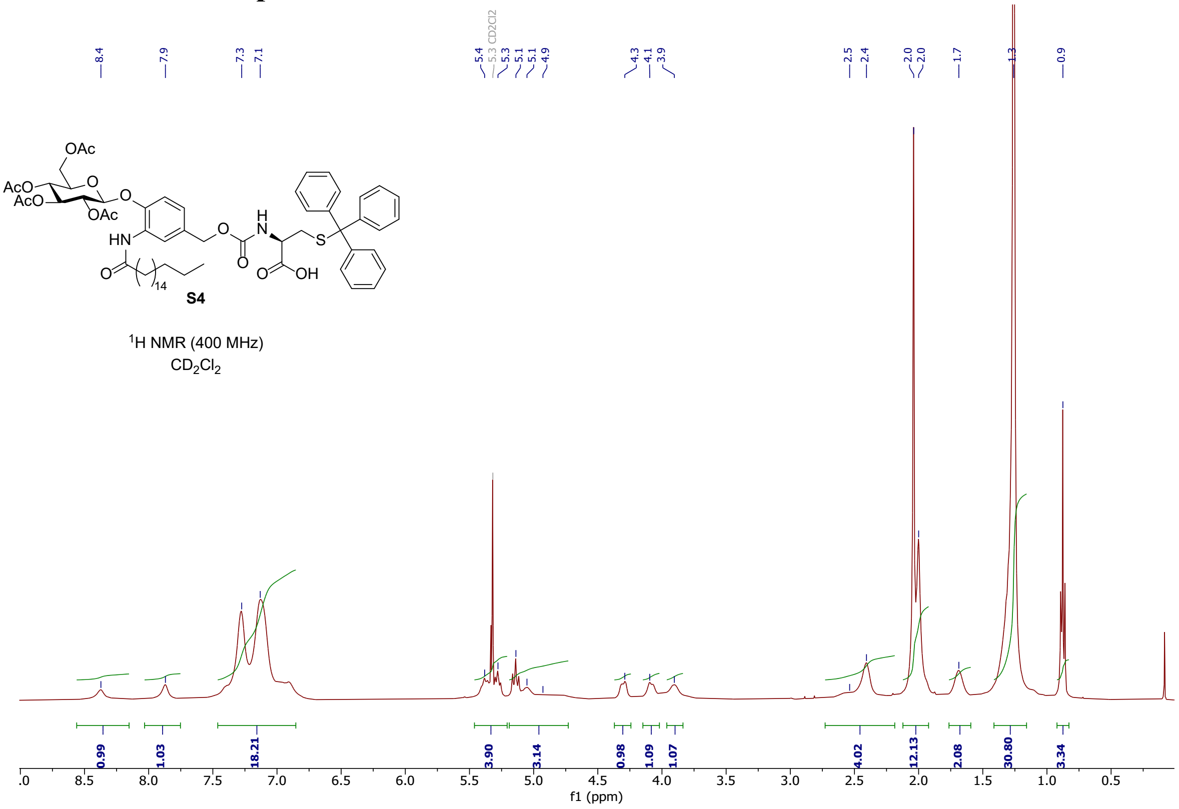


## 3.3.13 NMR compound 11

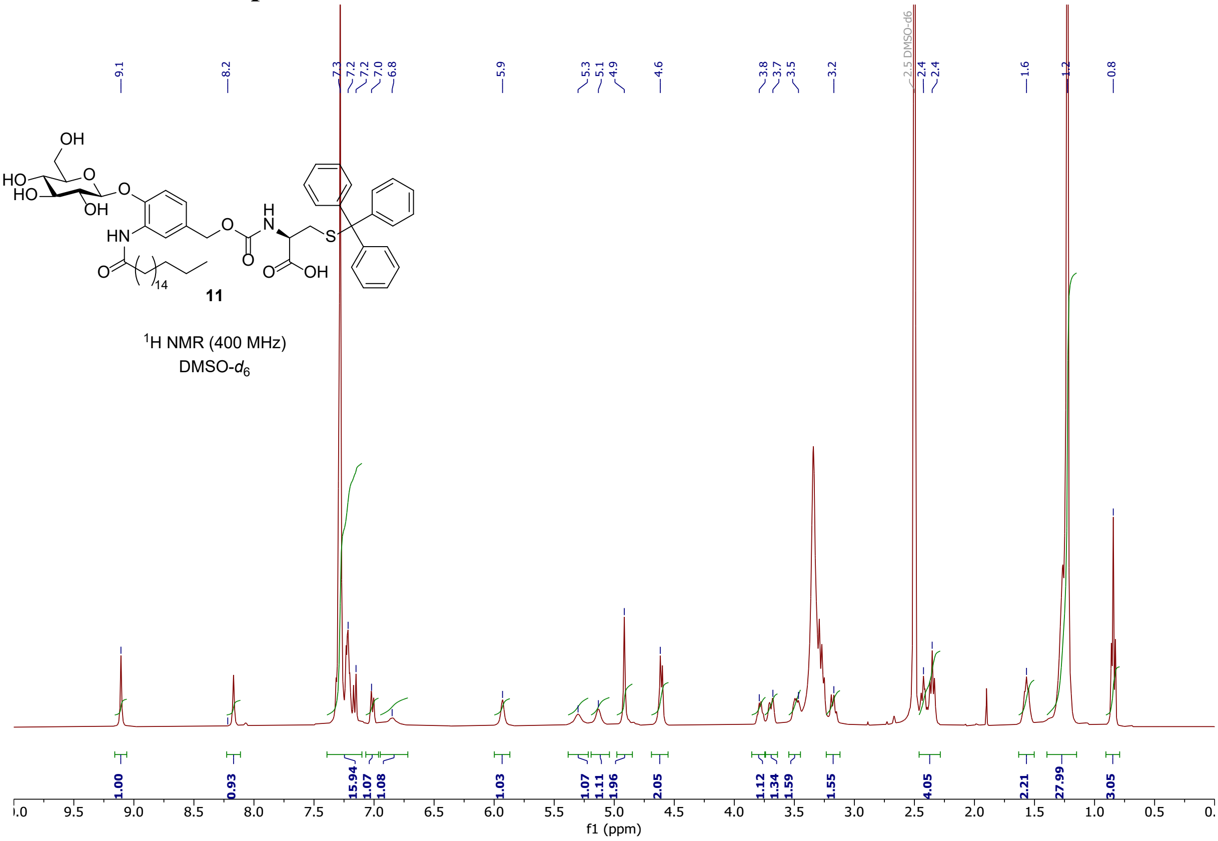

### 3.3.14 NMR compound S5

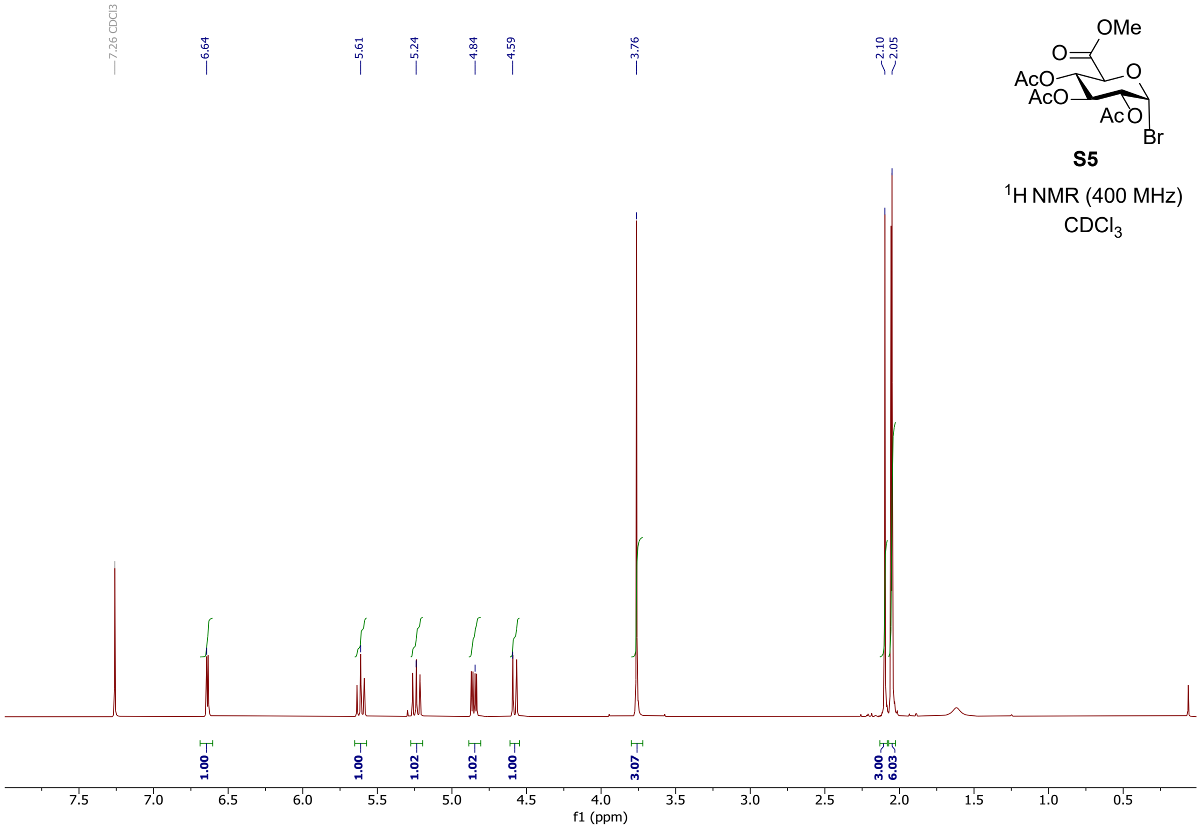


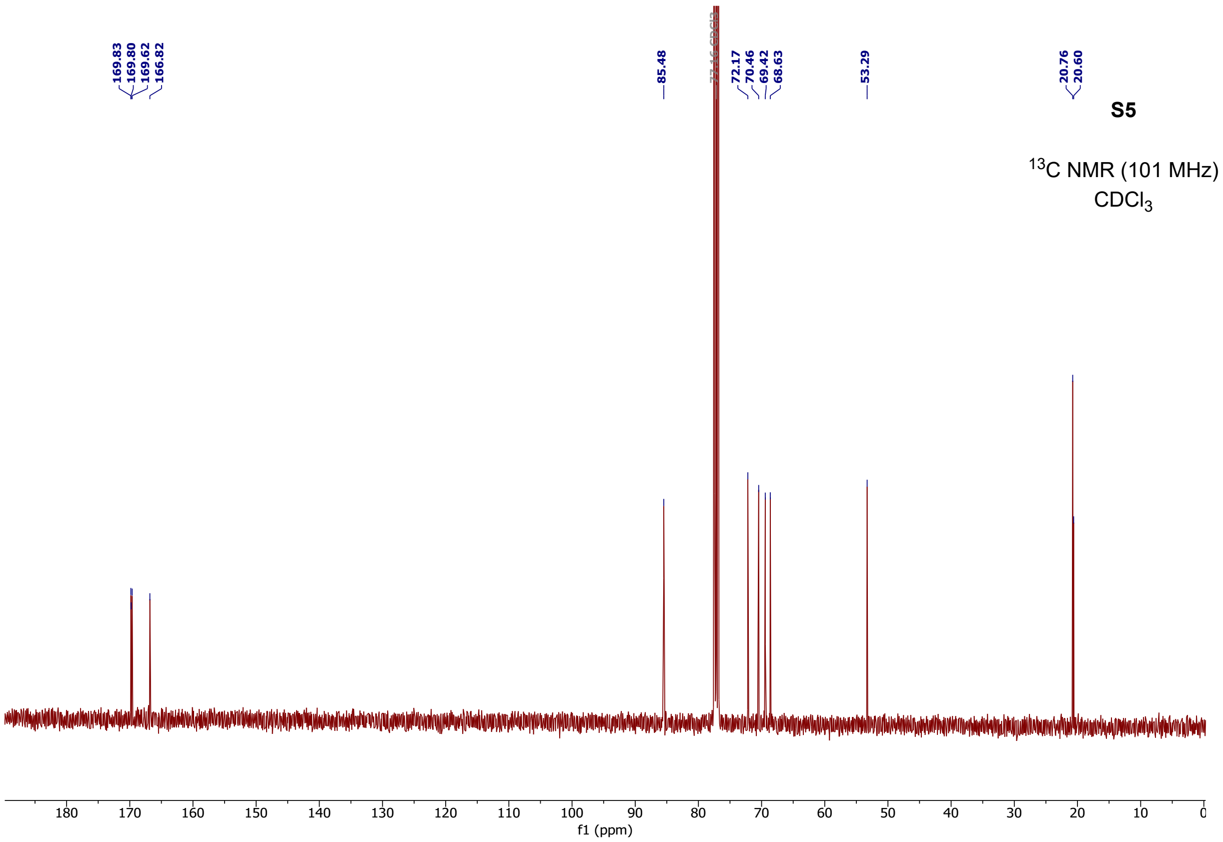

### 3.3.15 NMR compound 13

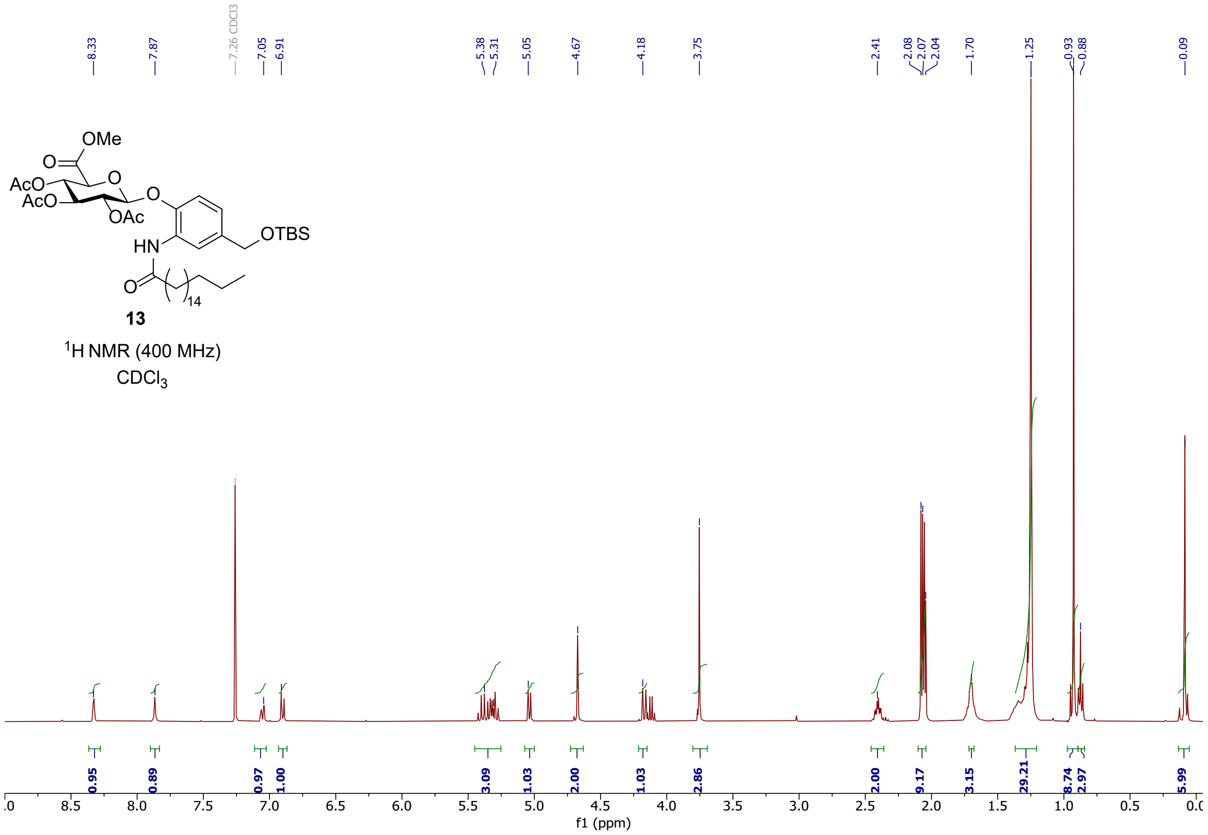


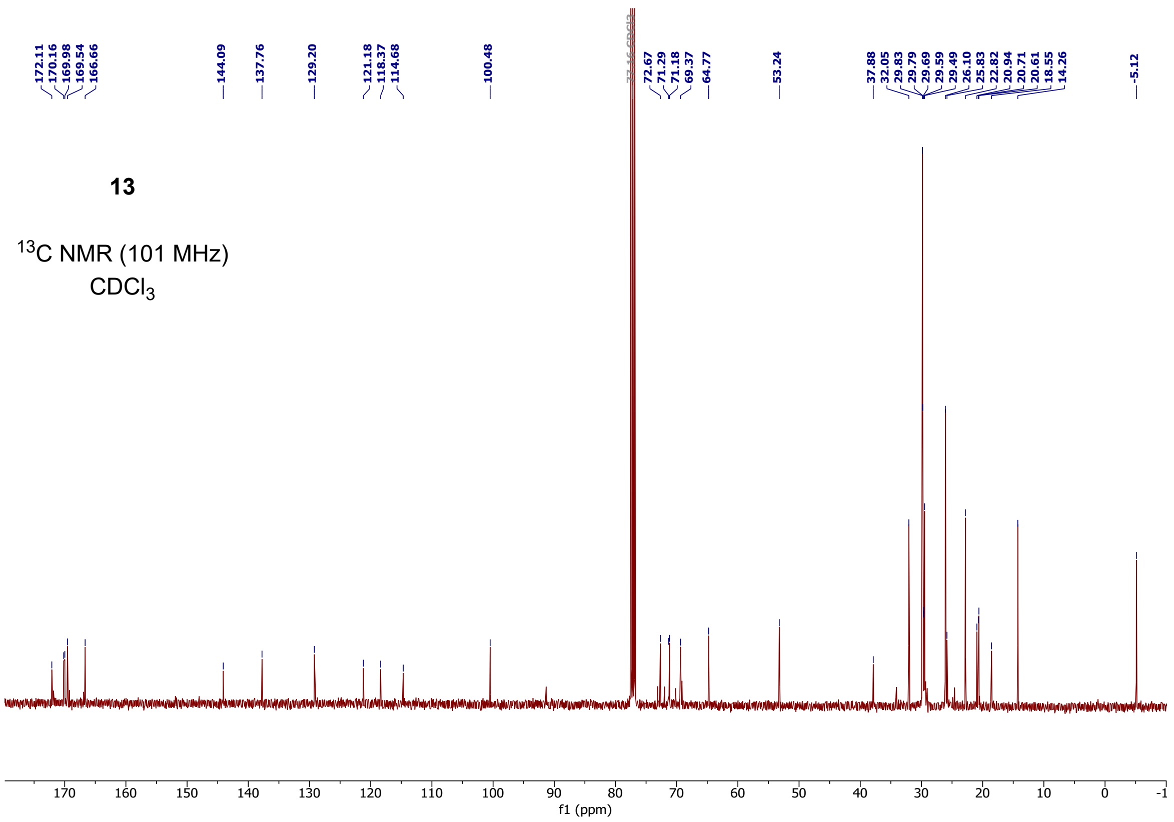

### 3.3.16 NMR compound S6

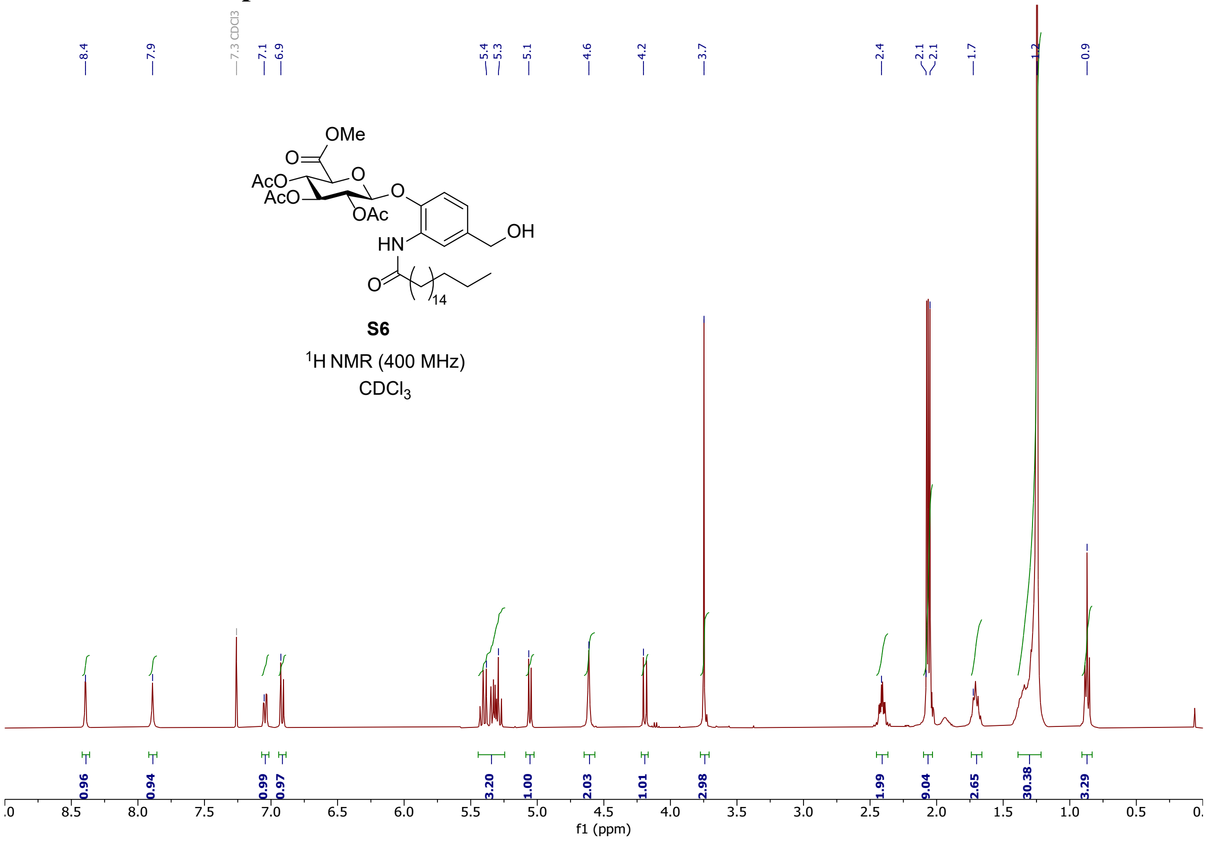


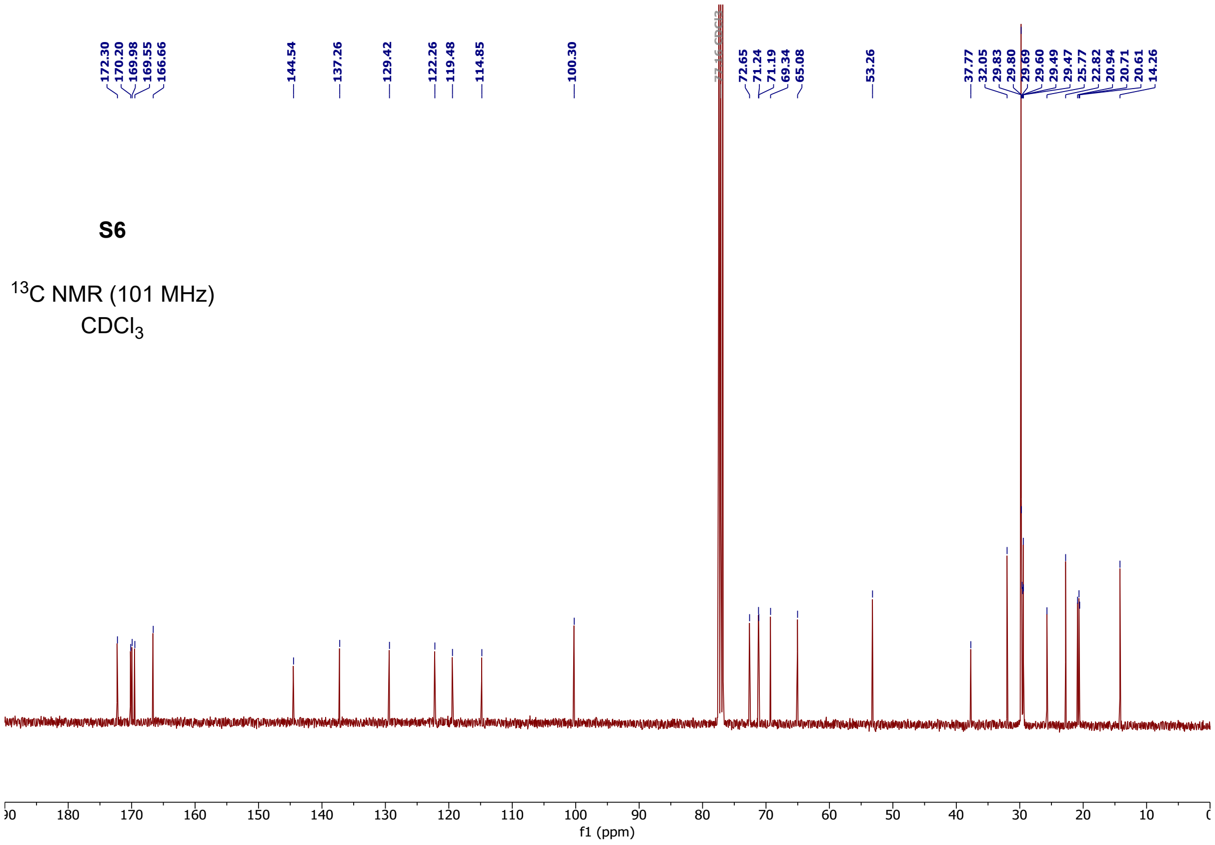

## 3.3.17 NMR compound S8

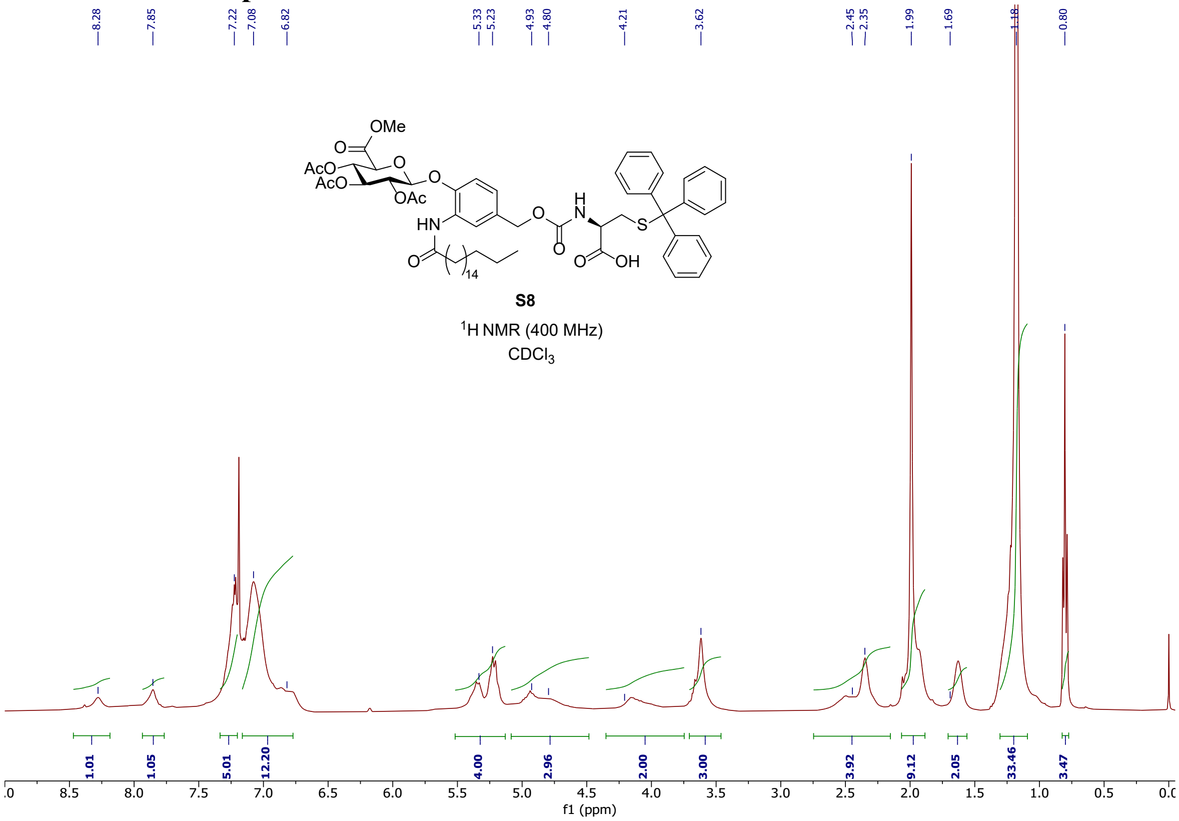


## 3.3.18 NMR compound 14

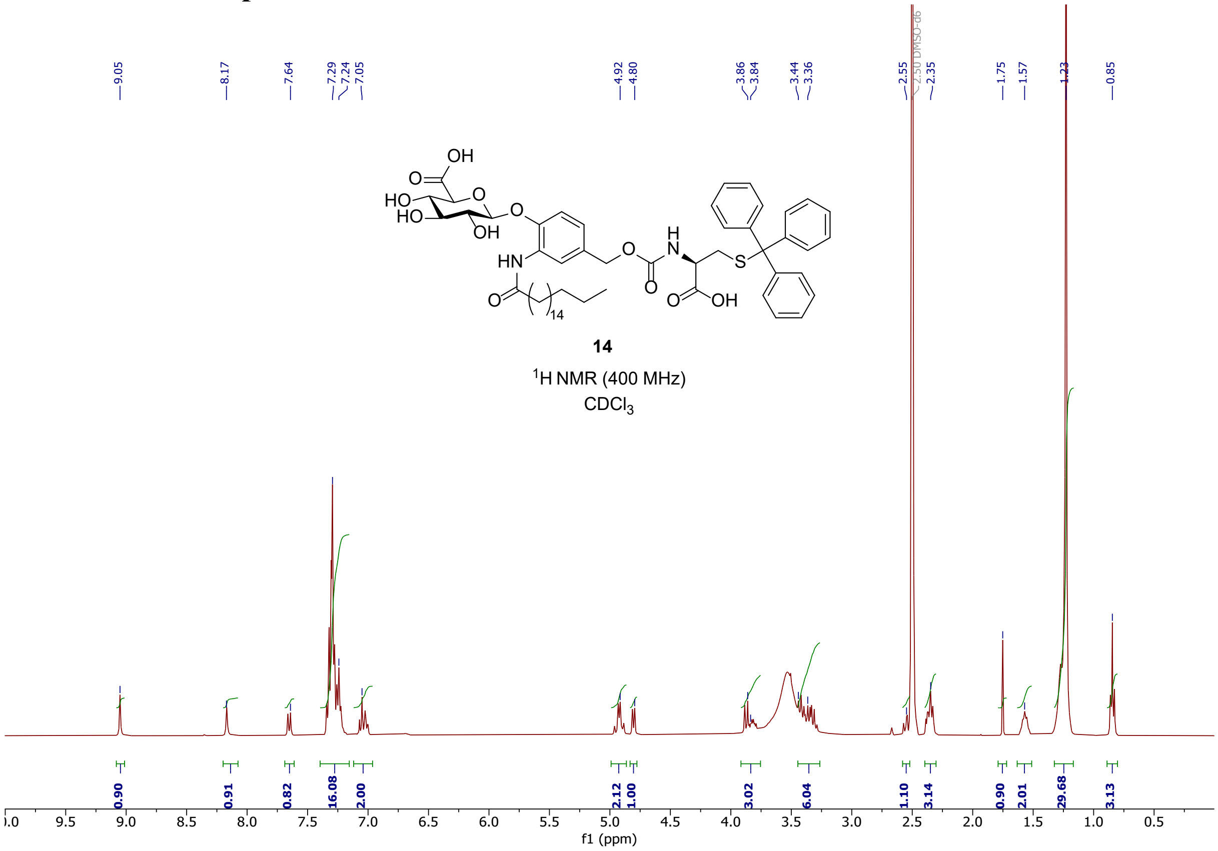

### 3.3.19 NMR compound S9

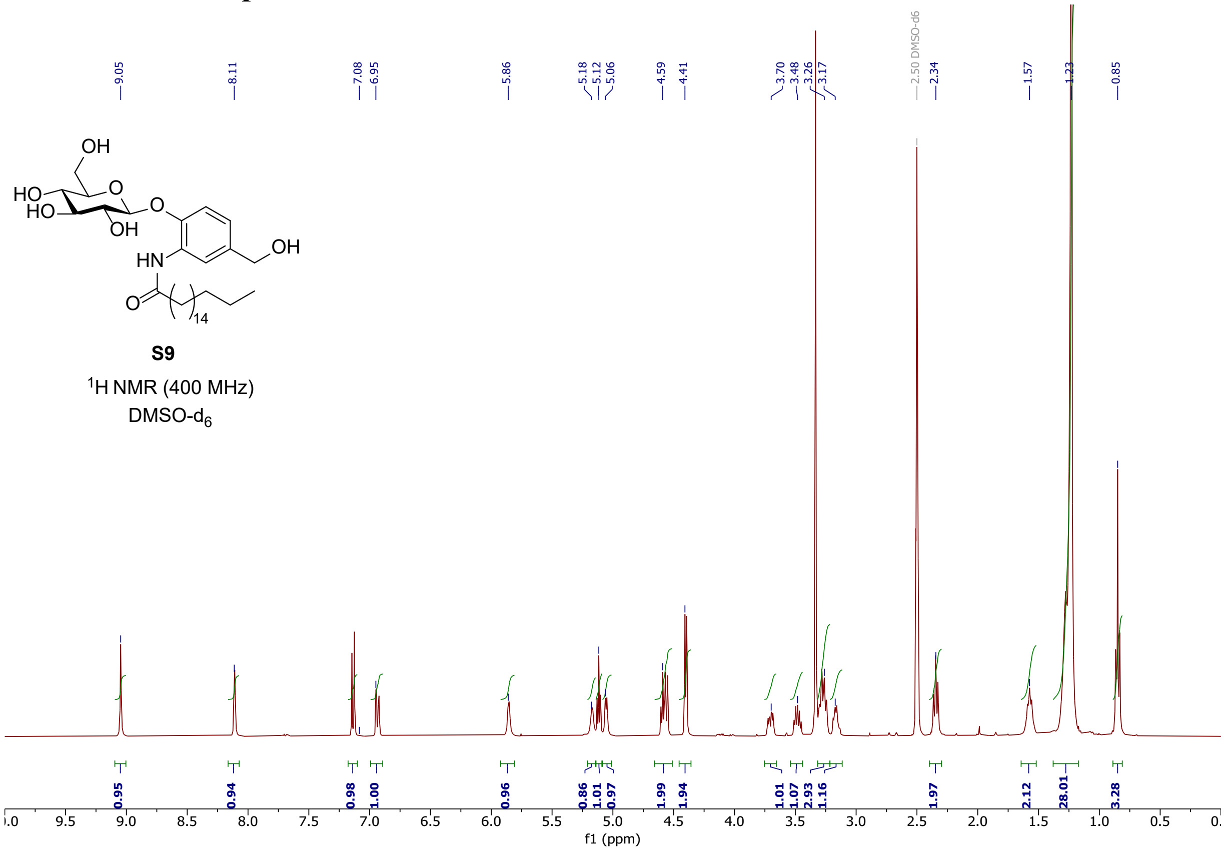


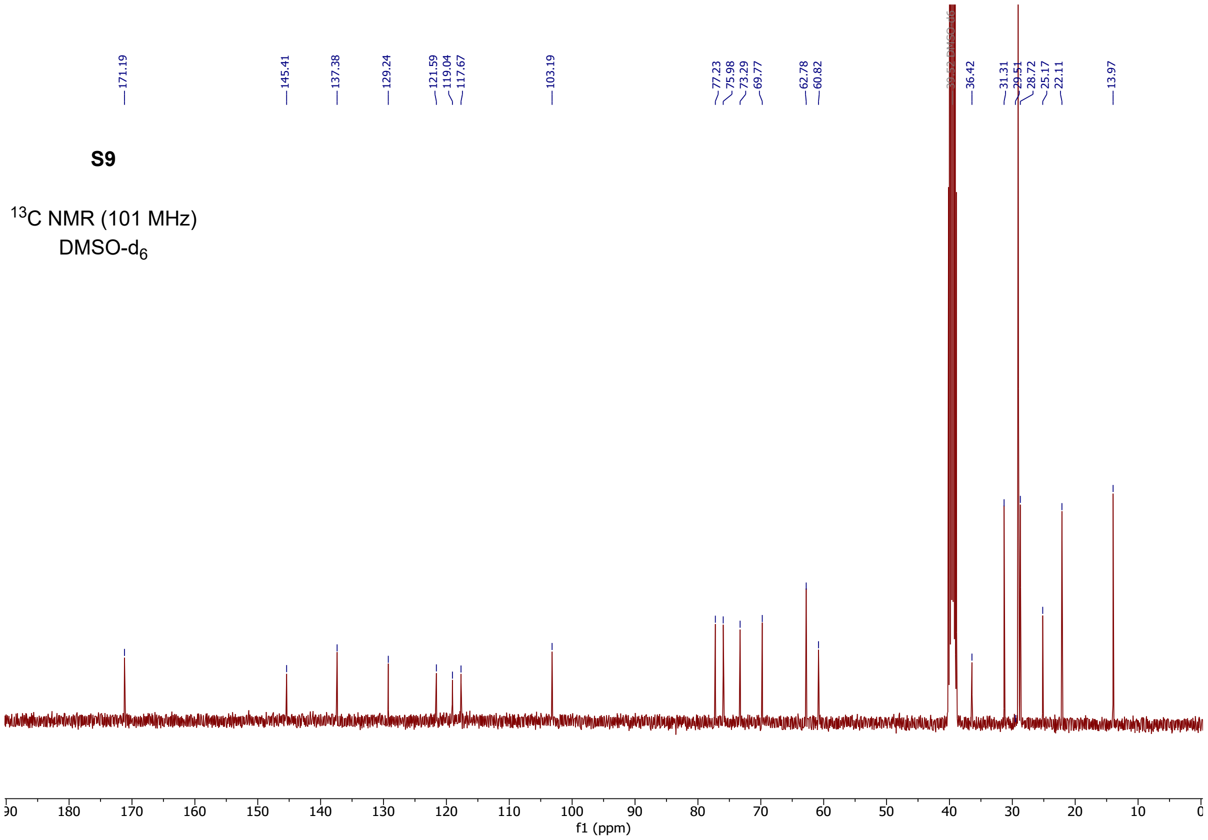

### 3.3.20 NMR compound S10

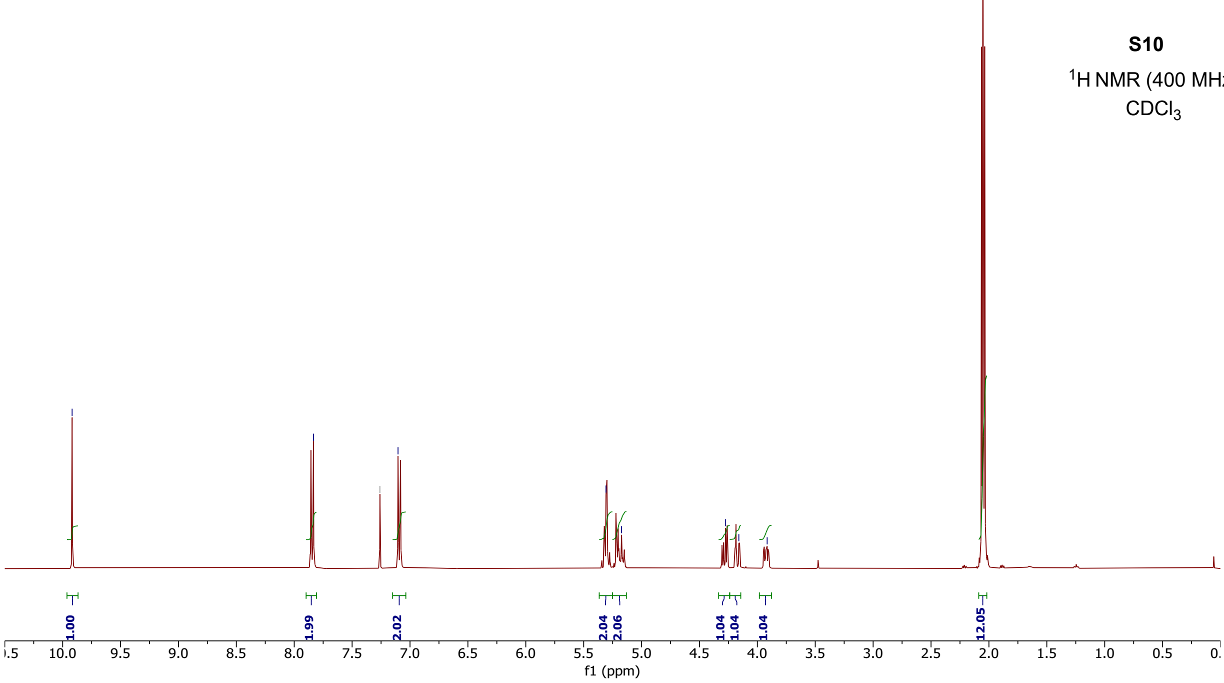


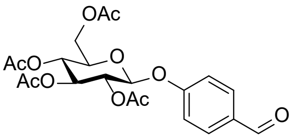


**S10**

$^{1}H$ NMR (400 MHz)
$CDCl_3$

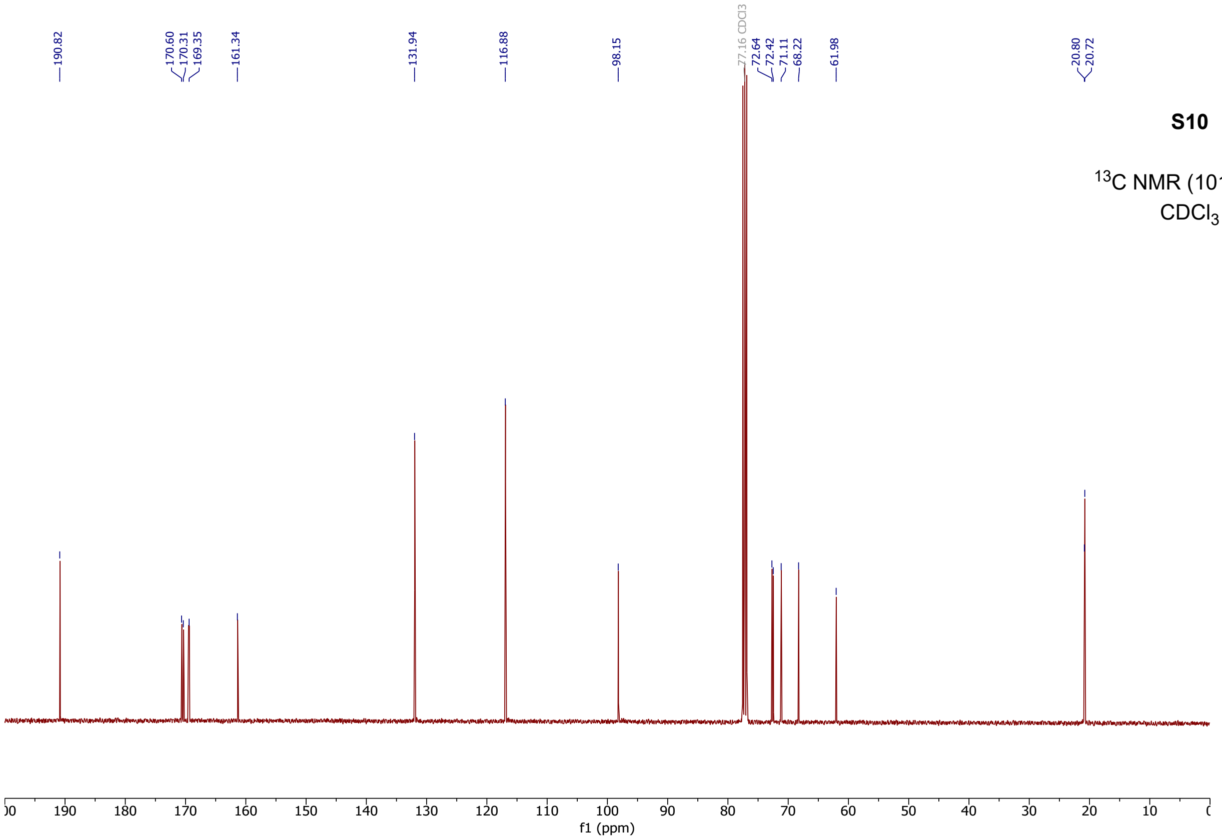


**S10**

$^{13}C$ NMR (101 MHz)
$CDCl_3$

### 3.3.21 NMR compound S11

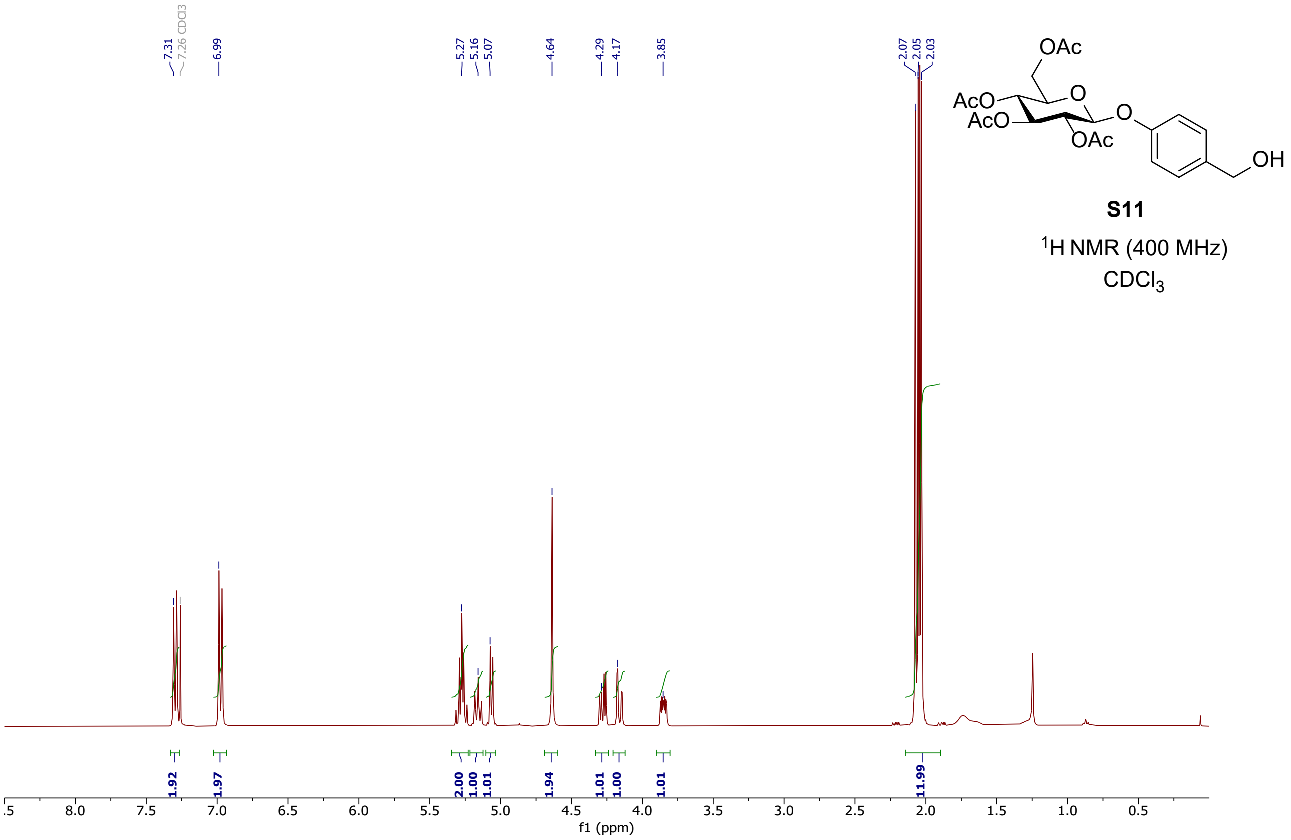


**S11**

$^{1}$H NMR (400 MHz)
$CDCl_3$

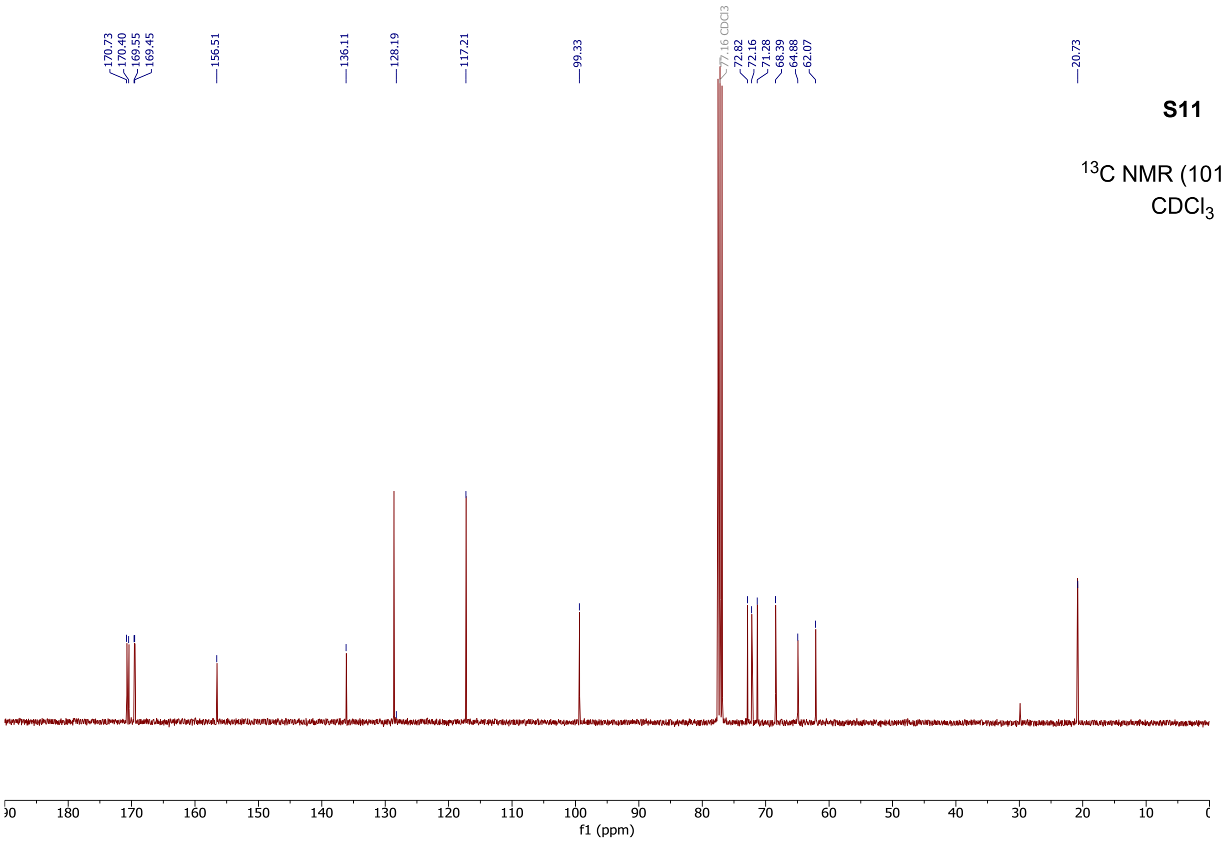


**S11**

$^{13}$C NMR (101 MHz)
$CDCl_3$

## 3.4 HPLC chromatograms

### 3.4.1 Phos-EAR (9)

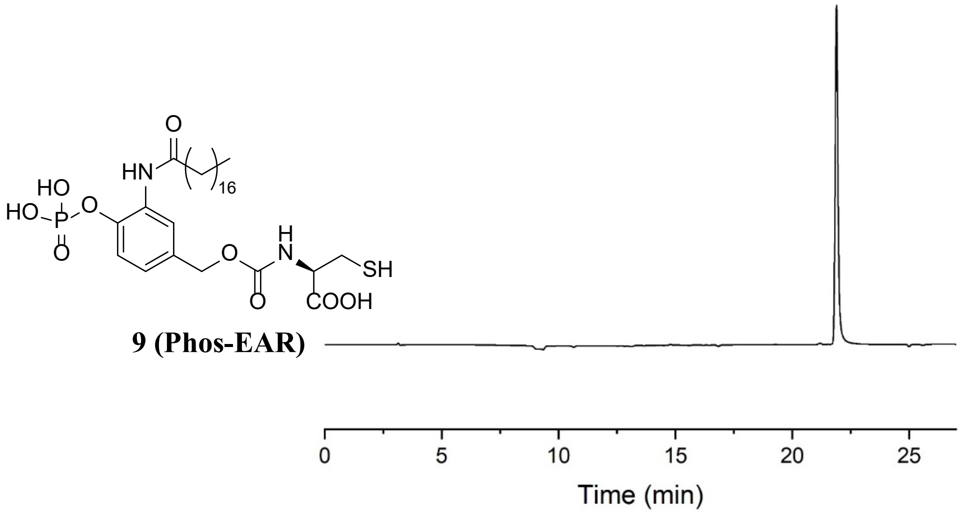


*HPLC chromatogram of **9 (Phos-EAR)** at 254 nm (**Analytical HPLC method A**)*

### 3.4.2 Glu-EAR (12)

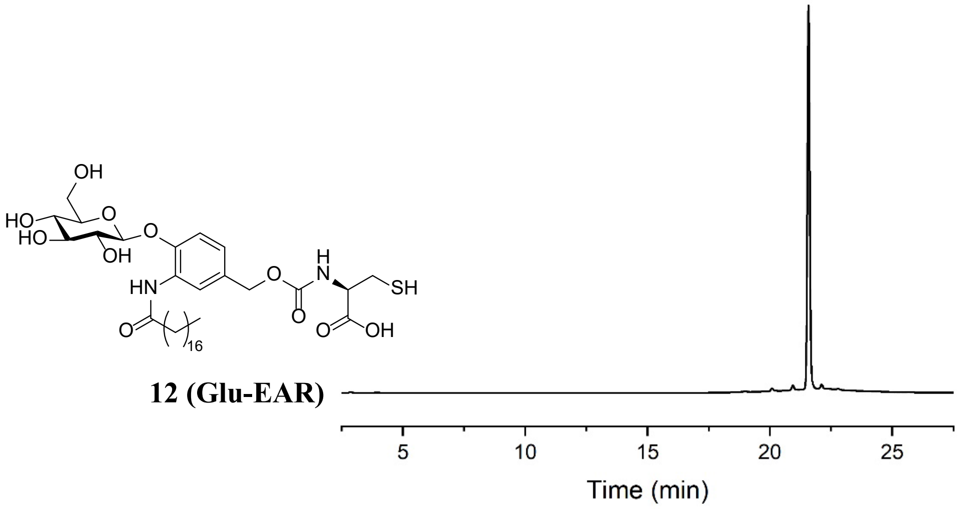


*HPLC chromatogram of **12 (Glu-EAR)** at 254 nm (**Analytical HPLC method B**)*

### 3.4.3 Gus-EAR (15)

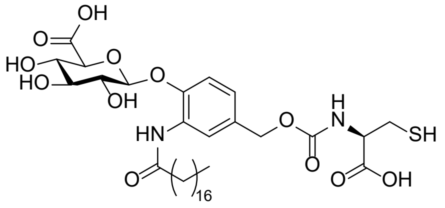


**15 (Gus-EAR)**

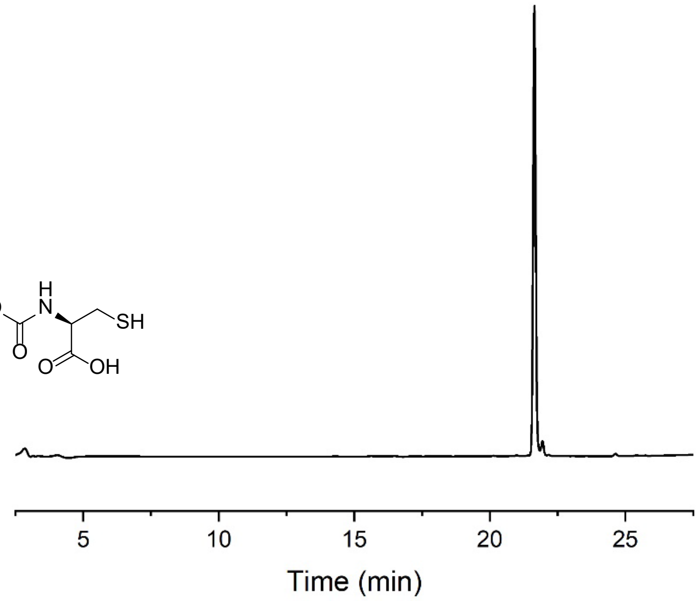


*HPLC chromatogram of **15 (Gus-EAR)** at 254 nm (**Analytical HPLC method B**)*
